# Loss-of-function mitochondrial DNA polymerase gamma variants cause vascular smooth muscle cells to secrete a diffusible mitogenic factor

**DOI:** 10.3389/fphys.2024.1488248

**Published:** 2025-02-17

**Authors:** Samantha Rothwell, Irvin Ng, Sophia Shalchy-Tabrizi, Pola Kalinowski, Omnia M. Taha, Italia Paris, Angelica Baniqued, Lisa Lin, Michelle M. Mezei, Anna Lehman, Lisa M. Julian, Damon Poburko

**Affiliations:** ^1^ Biomedical Physiology and Kinesiology, Simon Fraser University, Burnaby, BC, Canada; ^2^ Biological Sciences, Simon Fraser University, Burnaby, BC, Canada; ^3^ Molecular Biology and Biochemistry, Simon Fraser University, Burnaby, BC, Canada; ^4^ Centre for Cell Biology Development and Disease, Simon Fraser University, Burnaby, BC, Canada; ^5^ Institute for Neuroscience and Neurotechnology, Simon Fraser University, Burnaby, BC, Canada; ^6^ Adult Metabolic Diseases Unit, Vancouver General Hospital, Vancouver, BC, Canada; ^7^ Division of Neurology, University of British Columbia, Vancouver, BC, Canada; ^8^ Department of Medical Genetics, University of British Columbia, Vancouver, BC, Canada

**Keywords:** IncuCyte, StarDist, A7r5, mitochondrial DNA, hypertension, rare disease, POLG, vascular smooth muscle

## Abstract

**Introduction:**

Mitochondrial dysfunction promotes vascular aging and disease through diverse mechanisms beyond metabolic supply, including calcium and radical signaling and inflammation. Mitochondrial DNA (mtDNA) replication by the POLG-encoded mitochondrial DNA polymerase (POLG) is critical for mitochondrial health. Loss-of-function POLG variants are associated with a predisposition to hypertension. We hypothesized that impaired POLG, through reduced mtDNA copy number or other mechanisms, would promote smooth muscle hypertrophy or hyperplasia that drives vascular remodeling associated with hypertension.

**Methods:**

We characterized the effect of over-expressing POLG variants that were previously observed in a cohort of hypertensive patients (p.Tyr955Cys, p.Arg964Cys, p.Asn1098Ile, and p.Arg1138Cys) in A7r5 cells.

**Results:**

AlphaFold modeling of the POLG holoenzyme complexed with DNA predicted changes in the catalytic site in the p.Tyr955Cys and p.Asn1098Ile variants, while p.Arg964Cys and p.Arg1138Cys showed minimal effects. The POLG variants reduced mtDNA copy number, assessed by immunofluorescence and droplet digital PCR, by up to 27% in the order p.Tyr955Cys > p.Arg964Cys > p.Asn1098Ile > p.Arg1138Cys relative to wild-type-transfected cultures. Loss of mtDNA was reduced in cultures grown in low serum and glucose media, but the cell density was increased in the same rank order in both 10% serum and 1% serum. POLG constructs contained a Myc epitope, the counterstaining for which showed that the mtDNA copy number was reduced in both transfected cells and untransfected neighbors. Live-cell imaging of mitochondrial membrane potential with TMRM and radical oxygen species production with MitoSOX showed little effect of the POLG variants. POLG variants had little effect on oxygen consumption, assessed by Seahorse assay. Live-cell imaging growth analyses again showed increased growth in A7r5 cells transfected with p.Tyr955Cys but a decreased growth with p.Arg1138Cys, while p.Tyr955Cys increased growth of HeLa cells. Conditioned media from HeLa cells transfected with POLG variants reduced doubling times in naïve cultures. Pharmacologically, wedelolactone and MitoTEMPOL, but not indomethacin or PD98059, suppressed the mitogenic effects of p.Tyr955Cys and p.Arg964Cys in A7r5 cells.

**Discussion:**

We conclude that POLG dysfunction induces secretion of a mitogenic signal from A7r5 and HeLa cells even when changes in mtDNA copy number are below the limit of detection. Such mitogenic stimulation could stimulate hypertrophic remodeling that could contribute to drug-resistant hypertension in patient populations with loss-of-function POLG variants.

## 1 Introduction

Mitochondrial dysfunction is a significant contributor to the promotion of vascular aging and vascular dysfunction that can lead to hypertension. For example, aging and inflammation promote mitochondrial radical oxygen species (ROS) production, which is linked to disinhibition of MAPK signaling (Kulawiec et al., 2009; Dikalov and Ungvari, 2013), while the release of cell-free mitochondrial DNA (mtDNA) into the circulation in response to inflammatory and noxious stimuli is associated with TLR9 activation and inflammatory signaling (McCarthy et al., 2015; McCarthy et al., 2018). Aging blood vessels tend to have reduced mitochondrial DNA copy number (mCN), while mutations in mtDNA contribute to maternal heritance patterns of hypertension (Schwartz et al., 2004; Wilson et al., 2004; Ungvari et al., 2008; Liu et al., 2009; Elango et al., 2011). Consistent with the premise that mitochondrial dysfunction is associated with hypertension, we have reported that patients with two forms of mitochondria-associated genetic variants exhibited higher rates of hypertension than the age-matched general population (Hannah-Shmouni et al., 2014; Pauls et al., 2020). One group had high heteroplasmy levels for common m.3243A>G mitochondrial encephalopathy, lactic acidosis, and stroke-like episode (MELAS) mtDNA variant. The other group had chronic progressive external ophthalmoplegia (CPEO) associated with variants in the POLG gene, which encodes the DNA Polymerase Gamma, Catalytic Subunit (POLG) that is responsible for replicating mtDNA. In particular, the CPEO population with POLG variants had high rates of hypertension that were refractory to combined anti-hypertensive drug therapy, suggesting that POLG variants were associated with structural changes in blood vessels involving smooth muscle hypertrophy or hyperplasia, rather than inducing elevated vascular tone.

While numerous POLG variants are associated with neuromuscular disorders and several with tumorigenesis (Graziewicz et al., 2004; Wong et al., 2008; Singh et al., 2009; Singh et al., 2015), little is known about the impact of POLG variants on vascular function. In the vasculature, mitogenic signaling in contractile, quiescent smooth muscle cells can lead to polyploidy *via* endoreduplication, if cell division is suppressed, or neointimal proliferation, when mitotic stimulation is sufficient and prolonged (Hixon et al., 2000; Hixon and Gualberto, 2003; Owens et al., 2004). Downregulation of the mitochondrial fusion promoting protein mitofusin2 and active mitochondrial division are involved in and are reportedly necessary for smooth muscle proliferation and vascular remodeling (Chen et al., 2004; Chalmers et al., 2012). Similarly, increased mitophagy is a key step in smooth muscle phenotypic switching from contractile to proliferative states (Pearce, 2024). Whether or how genetically impaired POLG function influences smooth muscle function or phenotype is currently not known. We hypothesized that the association of POLG-related diseases with drug-resistant hypertension could suggest that POLG variants promote smooth muscle hypertrophy and or hyperplasia promoting vascular remodeling.

To assess the role of POLG pathogenic variants and variants of unknown significance (VUSs), we created a series of exogenous expression vectors encoding wild-type POLG or clinically observed variants and carried out overexpression of these variants in A7r5 smooth muscle cells or HeLa cells in culture. The variants selected included the pathogenic p.Tyr955Cys (c.2864A>G) and the p.Arg964Cys (c.2890C>T) variant that predisposes carriers to nucleoside reverse transcriptase inhibitor-induced mitochondrial toxicity (Graziewicz et al., 2004; Bailey et al., 2009; Estep and Johnson, 2011). Both variants are known to reduce polymerase activity and accuracy, where the Y955C exhibits the more severe phenotype (Ponamarev et al., 2002; Bailey et al., 2009). Notably, a homozygous p.Arg964Cys (c.2890C>T) variant was also attributed to juvenile liver failure in the absence of neurological symptoms (Kadohisa et al., 2024). We further selected two VUSs found in our cohort of CPEO patients: p.Asn1098Ile (c.3293A>T) and p.Arg1138Cys (c.3412C>T). These VUSs are located in the 1F subcluster of the POLG polymerase domain, and neither alone is predicted to be dominant or be likely to cause POLG-related symptoms alone by the POLG Pathogenicity Prediction Server (www.mitomap.org). However, p.Arg1138Cys was reported in cases of progressive external ophthalmoplegia and sensory ataxic neuropathy, dysarthria, and ophthalmoparesis in *trans* with p.Ala467Thr (Wong et al., 2008). A related p.Asn1098Lys variant was reported in a case of Alpers–Huttenlocher syndrome (Iodice et al., 2016). These reports suggest the pathological potential of the p.Arg1138Cys and p.Asn1098Ile variants, but their specific impacts on POLG function have not been reported previously. Using these variants, we assessed predictions of the POLG/DNA interaction with AlphaFold, cell proliferation with endpoint assays and live imaging, and analyses of mitochondrial membrane potential, radical oxygen species generation, and oxygen consumption. Subsequently, we refer to variants by single-letter abbreviations for compactness (i.e., Y955C, R964C, N1098I, and R1138C).

## 2 Methods

### 2.1 Chemical, reagents, and abbreviations

Chemicals and drugs were purchased from Sigma-Aldrich unless otherwise noted. Laminin (CACB354232) was purchased from VWR Scientific. Dulbecco’s modified Eagle medium (DMEM, cat# 10569-010 & 10567-014), fetal calf serum and antibiotics (15140-122), Hoechst-33342 (H3570), and Lipofectamine 3000 (L3000008) were sourced from Thermo Fisher Scientific. Paraformaldehyde (RT-15710) was obtained from Electron Microscopy Sciences (Hatfield, PA). Normal goat serum (G9023) was obtained from EMD Millipore. Dulbecco’s phosphate-buffered solution (PBS) (in mM: 2.67 KCl, 1.47 KH_2_PO_4_, 138 NaCl, and 8.1 NaH_2_PO_4_) was used. Additional reagents are described as they are used in the specific methods sections. Abbreviations: ΔΨ_m_: mitochondrial membrane potential, AA: antimycin A, ANOVA: analysis of variance, ACTB: beta actin, CPEO: chronic progressive external ophthalmoplegia, ddPCR: droplet digital PCR, DMEM: Dulbecco’s modified Eagle’s medium, DM: differentiation media, dsDNA: double-stranded DNA, ERK: extracellular signal-regulated kinase, EGFP: enhanced green fluorescence protein, FBS: fetal bovine serum, FCCP: carbonyl cyanide 4-(trifluoromethoxy)phenylhydrazone, Fiji: Fiji is just ImageJ, FOV: field of view, GFP: green fluorescent protein, GM: growth media, H2B-RFP: histone 2B red fluorescent protein, IKKα/ß: IkappaB kinase α/ß, MAPK: mitogen-activated protein kinase, mCN: mitochondria DNA copy number, MELAS: mitochondrial encephalomyopathy with lactic acidosis and stroke-like episodes, mtDNA: mitochondrial DNA, N1098I: POLG p.Asn1098Ile variant, NCBI: National Center for Biotechnology Information, ND1: mitochondrially encoded NADH dehydrogenase 1, NEB: New England Biolabs, NLS: nuclear localization sequence, OCR: oxygen consumption rate, Oligo: oligomycin A, PBS: phosphate-buffered solution, PCR: polymerase chain reaction, PFA: paraformaldehyde, POLG: polymerase gamma, R964C: POLG p.Arg964Cys variant, R1138C: POLG p.Arg1138Cys variant, ROI: region of interest, ROS: radical oxygen species, TLR9: toll-like receptor 9, TMRM: tetramethylrhodamine methyl ester, VUS: variant of unknown significance, WT: wild-type gene, and Y955C: POLG p.Tyr955Cys variant.

### 2.2 Cell culture and transfection

HeLa cells stably co-expressing a chromatin marker (H2B-RFP) were generously sourced from the Gerlich lab (Institute of Molecular Biotechnology of the Austrian Academy of Sciences) (Steigemann et al., 2009). A7r5 cells were purchased from the American Type Culture Collection (ATCC, CRL-1444). Prior to experiments with A7r5 and HeLa cells, cultures were tested for *mycoplasma* contamination with the *Mycoplasma* PCR Detection Kit (ABM, G238) and were confirmed to be mycoplasma-free. Cells were grown at 37°C in a humidified 5% CO_2_ incubator with antibiotic-free DMEM (Gibco, 10569-010) supplemented with 10% fetal bovine serum (FBS) (Seradigm, 89510-186 or Corning, 35-077-CV) for growth media (GM) (Kim et al., 2021). The A7r5 differentiation medium (DM) was the low-glucose (1 g/L) variation of DMEM (10567-014) supplemented with 1% FBS, as previously described (Kim et al., 2021). Stable transfection of HeLa-H2B-RFP was maintained with 0.5 μg/mL puromycin (ABM, G264) selection antibiotic, and HeLa cells were grown in GM or in the same media with 1% serum (DM). Cells were passaged at ∼80% confluency, and all experiments were performed between passages 5 and 25 for A7r5 and 11 and 36 for HeLa. Cells were seeded on multi-well plates, allowed to attach for 24 h, and then transfected the following day with 0.2 µg (96-well), 1 µg (24-well), or 3.75 µg (6-well) of DNA per well using a 1:1 ratio of Lipofectamine 3000 (Invitrogen, L3000-008) and a 1 µg: 2 µL DNA: P3000 ratio for 24 h (or 6 h for Figure 8) before the medium was replaced with fresh GM or DM. The number of replicate wells that were transfected is given in figure legends. To assess the impact of over-expression of wild-type POLG, in most experiments, untransfected wells were also observed as a form of negative control.

### 2.3 Molecular cloning

We introduced variants into the human POLG (NM_002693) coding sequence on a pCMV6 backbone (RC204456, OriGene) using the Q5^®^ Site-Directed Mutagenesis Kit (E0552S, New England Biolabs (NEB)). First, 12.5 µL of the master mix was combined with ∼1 ng of plasmid DNA and 10 µM forward and reverse primers (see Table 1) with nuclear-free water to make up the volume to 25 µL, followed by thermocycling at 98°C 30 s (activate hot start polymerase), and then 25 cycles of 98°C 10 s, Tm 30 s, 72°C 300 s (30 s/kb) and then 72°C for 120 s. The PCR product (1 µL) was combined with 5 µL of 2x KLD (kinase, ligase, and DpnI) buffer, 1 µL of KLD enzyme mix, and 3 µL of nuclease-free water, which was mixed by trituration and incubated at room temperature for 5 min before being transformed into high-efficiency NEB 5-alpha competent *E. coli* by heat shock treatment and plating on kanamycin+ (50 μg/mL) agar plates. Plasmid DNA was extracted from 2 to 4 colonies (Qiagen DNEasy miniprep) and sent for Sanger sequencing (Genewiz). We amplified two mutant colonies to create plasmid stocks with an endotoxin-removing Midiprep kit (E.Z.N.A.^®^ Endo-Free Plasmid DNA Midi Kit Cat #D6915-03 or Promega PureYield Midiprep Cat # PR-A2492).

**TABLE 1:** Cloning primers.

Description	Sequence (5’→3′)	Tm (^o^C)
ddPCR		
ND1 (rat)	F: TTAACGTCGAATACGCCGCA R: AGCTGGTTGAGTATAATTCAGGGT	56.0
ACTB (rat)	F: GGGATGTTTGCTCCAACCAA R: GCGCTTTTGACTCAAGGATTT	56.0
POINT MUTATIONS		
POLG c.2864A > G (Y955)	F: GGCCGCATCTgTGGTGCTGGG R: GTAGTTGAAGATTTTGGCATGCTCACG	65.0
POLG c.2890C > T (R964C)	F: CTTTGCTGAGtGCTTACTAATGCAG R: GGCTGCCCAGCACCATAG	65.0
POLG c.3293A > T (N1098I)	F: AGCCGTGTGAtTTGGGTGGTA R: GGTCATAAACTCTTCCTGGACAGC	65.0
POLG c.3412C > T (R1138C)	F: TGACGAGGTTtGCTACCTGGT R: TGGATGCTGATGCAGAAGCG	62.8
pPOLG::T2AP2A:3xNLS-EGFP FRAGMENTS		
POLG (CDS from RC204456 and variants)	F: GGAATTCGTCGACTGGAT R: AACCTTATCGTCGTCATCCT	64.7
pEGFPexNLS (backbone)	F: GTCGCCACCATGGTGAGCAA R: AGCGGATCTGACGGTTCA	64.0
P2AT2A (Addgene 87,829)	F: GGAGGCAGAAAGCTTGGT R: CTCGGTAGGTCCAGGATTCT	66.0
pPOLG::T2AP2A:3xNLS-EGFP GIBSON ASSEMBLY		
POLG	AGTGAACCGTCAGATCCGCTGGGAATTCGTCGACTGGAT GAACCAAGCTTTCTGCCTCCAACCTTATCGTCGTCATCCT	72.0
pEGFP3xNLS	AGAATCCTGGACCTACCGAGATGGTGAGCAAGGGCGAGGA GATCCAGTCGACGAATTCCCAGCGGATCTGACGGTTCA	72.0
P2AT2A	AGGATGACGACGATAAGGTTGGAGGCAGAAAGCTTGGT TCCTCGCCCTTGCTCACCATCTCGGTAGGTCCAGGATTCT	72.0

To create POLG-expression constructs with self-cleaving nuclear-targeted EGPF, we cloned the POLG (or variants) coding sequences and a P2AT2A (Addgene 87829) sequence 5′ of the EGFP3xNLS on pEGFP-C1 EGFP-3XNLS (Addgene 58468) by Gibson assembly. The POLG sequence, P2AT2A sequence, and pEGFP-C1 EGFP-3xNLS backbone were separately amplified with primers (10 µM) designed using NCBI’s primer blast (Table 1) and Q5^®^ Hot Start High-Fidelity 2X Master Mix (M0494S) in 25 µL reactions using 10 ng of the template and 34 cycles of the annealing temperatures listed in Table 1. Amplicons were verified for the correct size by agarose gel electrophoresis and sequenced by Sanger sequencing (Genewiz). We amplified these amplicons with primers containing overlapping sequences for Gibson assembly (Table 1) and combined the column purified fragments at a ratio of 1:1:5 (backbone: POLG: P2AT2A) using NEB’s Gibson Assembly Master Mix (E2611S). Bacterial transformation, sequence verification, and plasmid amplification were performed as described for site-directed mutagenesis.

### 2.4 IncuCyte live-cell imaging

We plated cells on clear 24-well plates or Greiner 96-well plates (655 090) as illustrated in the relevant figures and loaded cells with either 0.5 µM SiR-DNA (Cytoskeleton, CY-SC007) or a 3000x dilution of SPY-650 DNA (Cytoskeleton, CY-SC501) 2–3 h before beginning multi-day imaging with an IncuCyte (Sartorius, 2022A Rev1) imaging system. We collected images at ×10 magnification (1.24 µm/pixel) with green (GFP), orange (H2B-RFP), and near infra-red (far-red nuclear dyes) filters. Imaging continued for 2–3 days at 20- to 30-min intervals. After live-cell imaging was complete, we fixed cells with 4% paraformaldehyde (PFA) and washed and stored them in phosphate-buffered solution (PBS) for future imaging to determine the transfection efficiency. Uncalibrated 16-bit images were exported, compiled into time lapse stacks in Fiji, the nuclei were segmented using StarDist, and we used an ImageJ macro to extract nuclear intensity information for all imaged channels. HeLa cells were segmented with the Versatile Fluorescent Nuclei model, and A7r5 cells were segmented with a custom-trained model based on the Versatile Fluorescent Nuclei model. Nuclear ROIs were filtered for the minimal and maximal size and intensity, and ROIs passing these criteria were summarized at the level of the number of cells per FOV per frame using a custom-written JMP script that fit the growth curves to a 3-parameter exponential growth model (cell number = plateau x *e* ^kt^), where t = time, k = growth rate, and doubling time is then ln (2)/k.

### 2.5 Nikon live-cell and fixed imaging

We imaged multi-well plates and slides with a motorized Nikon TiE microscope with an Andor Zyla 5.5 sCMOS camera, Sutter Lambda XL light source, and Lambda 10-3 shutters and filter wheels. Dyes were selectively imaged with a quad-band Sedat filter set with excitation and emission filters on separate wheels and a quad-band dichroic (DA/FI/TR/Cy5-4X4M-C-000, Semrock) (Kim et al., 2021). Imaging bands are optimized for Hoechst, GFP/Alexa488, TMRM/Alexa555/MitoSOX, and Alex647. The Nikon Imaging Systems (NIS) Elements software (v4.51.01) controlled image acquisition using Nikon’s Jobs to automate multi-well plate imaging. We imaged cells with a 10 × 0.4 NA objective or a ×20 Super Fluor 0.75 NA objective. Over the course of our experiments, cells were seeded at densities ranging from 1,000–4,000 cells per well on a 96-well plate (29–118 cells/mm^2^). For TMRM and MitoSOX experiments, cells were plated at 7,000 cells per well. For analyses of drug treatment on POLG’s mitogenic effects, cells were plated at 2,000 cells per well. For drug treatments, drugs were diluted at least 1,000-fold from stock solutions dissolved in water [indomethacin (I7378, Sigma-Aldrich), MitoTempol (ab144644, Abcam)] or DMSO [wedelolactone (W4016, Sigma-Aldrich), PD 98059 (513000, EMD Millipore)]. Vehicle controls were not included as growth effects of POLG variants were compared to wild-type transfected cells.

### 2.6 Immunocytochemistry

A7r5 cells were plated on 96-well plates (Greiner, 655090) at 1,000–4,000 cells/well or glass coverslips (Fisher Scientific, 12-545-80) treated with 1 M hydrochloric acid (Sigma-Aldrich, 258148) and coated with 50 μg/mL Poly-L-lysine (MilliporeSigma, P2636) and 10 μg/mL laminin (Corning, 354232) that were plated in 24-well plates at 50,000 cells/well. Cells were transfected and assayed 48–72 h after removal of transfection mixtures. We fixed cells with 4% PFA in PBS and permeabilized with 0.1% Triton X-100 (Sigma, T8787-100 mL) and blocked with 2% goat serum (EMD Millipore S-26-100 mL) with 1% bovine serum albumin (Sigma, A-7960) before being stained with anti-DNA (Millipore, CBL186, 1:300) and/or anti-Myc (4A6 clone, EMD Millipore 05-724, 1:500-1:1000) or anti-Flag M2 (Sigma, 1804, 1:500-1:1000) overnight at 4°C. Primary antibodies were labeled with 1:1000 dilutions of goat-anti-mouse IgM Alexa 488 (Thermo, A21042) or 1:1000 dilution of goat-anti-mouse IgG1 Alexa-647 (Thermo, A21240).

### 2.7 Mitochondrial membrane potential and mitochondrial ROS production

We plated cells on 96-well plates at 6,000 cells/well and transfected them with 0.15–0.20 µg DNA (pPOLG:P2AT2A:EGFP3xNLS or the Y955C or R964C variants) the following day with a 1:1 ratio of Lipofectamine 3000, as mentioned above. Additional wells were not transfected as negative controls. Media was replaced with fresh GM the following day (∼24 h post-transfection), and cells were stained and imaged ∼48 h post-transfection. We labeled cells with 25–50 nM tetramethylrhodamine methyl ester (TMRM) (Thermo, T6668) and 3 μg/mL Hoechst 33342 (Thermo, H3570) in HEPES-buffered saline (in mM: 140 NaCl, 5 KCl, 2 CaCl_2_, 1.5 MgCl_2_, 10 glucose, and 10 HEPES, at pH 7.4) for 30 min in a 37°C cell culture incubator prior to imaging. Preliminary experiments indicated that TMRM quenching was not evident below 300 nM TMRM (not shown). We treated cells with 0.3 µM carbonyl cyanide 4-(trifluoromethoxy)phenylhydrazone (FCCP) (Sigma, C2920) or 10 µM oligomycin (Sigma, O4876), as indicated in figures. Drugs were present during pre-labeling and imaging. We measured mitochondrial ROS in parallel with mitochondrial membrane potential on additional 96-well plates, while labeling cells with 5 µM MitoSOX (Thermo, M36008) in HEPES-buffered saline for 30 min before imaging. We included 1 µM antimycin A (Sigma, A8674) at the time of dye addition. We imaged plates on the Nikon TiE microscope with the ×20 Super Fluor objective in a microscope enclosure that was heated to 30–32°C. We automatically acquired six predefined, unbiased fields of view in quadruplicate wells using the Nikon Perfect Focus system and NIS Elements Jobs for automated acquisition. The fluorescence intensity was analyzed as described in the *Image Analysis* section. Briefly, we created nuclear regions of interest based on intensity thresholds and expanded these ROIs to the lesser of 133 µm or equidistant boarders (i.e., Voronoi tessellation boundaries) from nuclear edges. In preliminary experiments, cells were imaged every 30 min for 5 h, and results in the first 30 min were comparable to those at 5 hours.

### 2.8 Seahorse

We determined the optimal plating density range in preliminary experiments (Supplementary Figure S4). For POLG analyses, A7r5 cells were plated in 6-well plates at 250,000–300,000 cells/well in GM and transfected the next day (pPOLG:P2AT2A:EGFP3xNLS or the Y955C or R964C variants) to achieve high transfection efficiency, while minimizing cell death. At 24-h post-transfection, the cells were passaged from the 6-well plate into uncoated Seahorse XF96 microplates (Agilent, 101085-004) at a density of 10,000 cells/well in GM or DM supplemented with CloneR2 (STEMCELL Technologies, 100-0691) to support cell survival and attachment. After 2 days of growth, cells were changed to Seahorse XF DMEM assay medium (Agilent Technologies, 103575-100) supplemented with 10 mM glucose, 1 mM pyruvate, and 2 mM L-glutamine and incubated for 1 hour in a 37°C non-CO_2_ incubator. We assessed the oxygen consumption rate (OCR) using the Seahorse XF Cell Mito Stress Test (Agilent, 103015-100) on a XFe96 Extracellular Flux Analyzer (Agilent) at 3-min increments starting at baseline, followed by the sequential addition of 1 µM oligomycin, 1 µM FCCP, and 1 µM rotenone/antimycin-A. The cells were then fixed with 4% PFA, stained with 3 μg/mL Hoechst-33342, and imaged at five locations per well with a ×20 objective on the Nikon TiE microscope to normalize the OCR per cell.

### 2.9 Image analysis

We performed all image analyses using Fiji (Schindelin et al., 2012). For Nikon-acquired images, automated cell segmentation employed threshold-based selection of Hoechst-labeled nuclei and expansion of nuclear ROIs by Voronoi tessellation, as we have previously described (Kim et al., 2021). We employed our custom Fiji macro roiBatchAnalysis_v3.8.ijm (https://github.com/dpoburko/ImageJ_macros/blob/master/roiBatchAnalysis_v3.8.ijm). We used a rolling background subtraction with a radius 1.5 times the largest expected nuclear diameter (∼50 µm for A7r5) and applied a single-intensity threshold for all images acquired in a single set-up staining that captured the maximal number of nuclei, while minimizing the coalescence of neighboring nuclei. We created ROIs surrounding but not including cell nuclei by dilating nuclear ROIs and excluding the inner nuclear ROI with the maximum dilation described in the *Results* section. Where basal background intensity needed to be ignored, the macro analyzed 32-bit images with pixels below a user-defined threshold set to not-a-number. For a batch (folder) of images, all ROIs for each cell (based on its nucleus) are saved to a single row of a .csv file for downstream analysis in JMP. For movies generated from the IncuCyte, we generated nuclear ROIs using the StarDist plugin for Fiji (Schmidt et al., 2018; Weigert and Schmidt, 2022), which was implemented using a custom-written macro to analyze folders of multi-frame images.

### 2.10 Droplet digital PCR

We performed droplet digital PCR (ddPCR) as described in Li et al. (2018). We extracted total genomic DNA from A7r5 and HeLa cells with a DNeasy Blood & Tissue Kit (Qiagen, 69504). PCRs contained 2–4 ng of genomic DNA, 1 µL of HindIII restriction enzyme (Thermo, FD0504), 2 µL of pre-mixed forward and reverse primers for each target at 12.5x the final concentration, 12.5 µL QX200 ddPCR EvaGreen Supermix (Bio-Rad, #1864034), and DNAse-free water to make up 25-µL reactions. Primer specificity was assessed by UCSC’s *in silico* PCR server (https://genome.ucsc.edu/cgi-bin/hgPcr) and by the amplification of a single population of droplet intensity. The final concentration of primers was 25 nM for ND1 and 180 nM for ACTB. See Table 1 for primer sequences. We cycled reactions in a C1000 touch thermocycler (Bio-Rad) using the protocol: 95°C for 5 min (HotStart), 40 cycles of 95°C for 30 s → 54°C for 90 s, 4°C for 5 min (stabilization), 90°C for 5 min (inactivation), and hold at 4°C. Reaction plates were cooled to room temperature or kept at 4°C overnight before reading on the QX200 droplet reader. For duplex reactions, positive droplets were gated in the 2D view using Quantsoft (v1.7).

### 2.11 Data processing, statistical analysis, and figure generation

Numerical data were compiled and analyzed using JMP^®^ (version 17.0.0, SAS Institute Inc., Cary, NC, 1989–2023). We used JMP’s ANOVA platform to perform one-way and factorial ANOVAs, with Dunnett *post hoc* tests to compare means against a control level, Tukey’s Honest Significant Difference test for adjusted, pair-wise comparisons, and linear contrasts (with Bonferroni adjustment) for comparisons of specific means in factorial ANOVAs. Results of ANOVAs in the text report least square mean values ± standard error. Figures show individual data points, with means shown as the thick, colored line and box plots with median (line), quartiles (box), and 1.5 times the interquartile range. No outliers were removed from the analyses. Outliers are shown unless noted in figure legends. Graphics and figures were compiled in PowerPoint (Microsoft), with cartoon illustrations created in BioRender. 3D models of proteins and DNA were created in the PyMOL molecular graphics system (v 2.5.4, Schrodinger). The number of experimental replicates is described in figure legends.

## 3 Results

### 3.1 Location of variants relative to pathogenic variants

**FIGURE 1:**
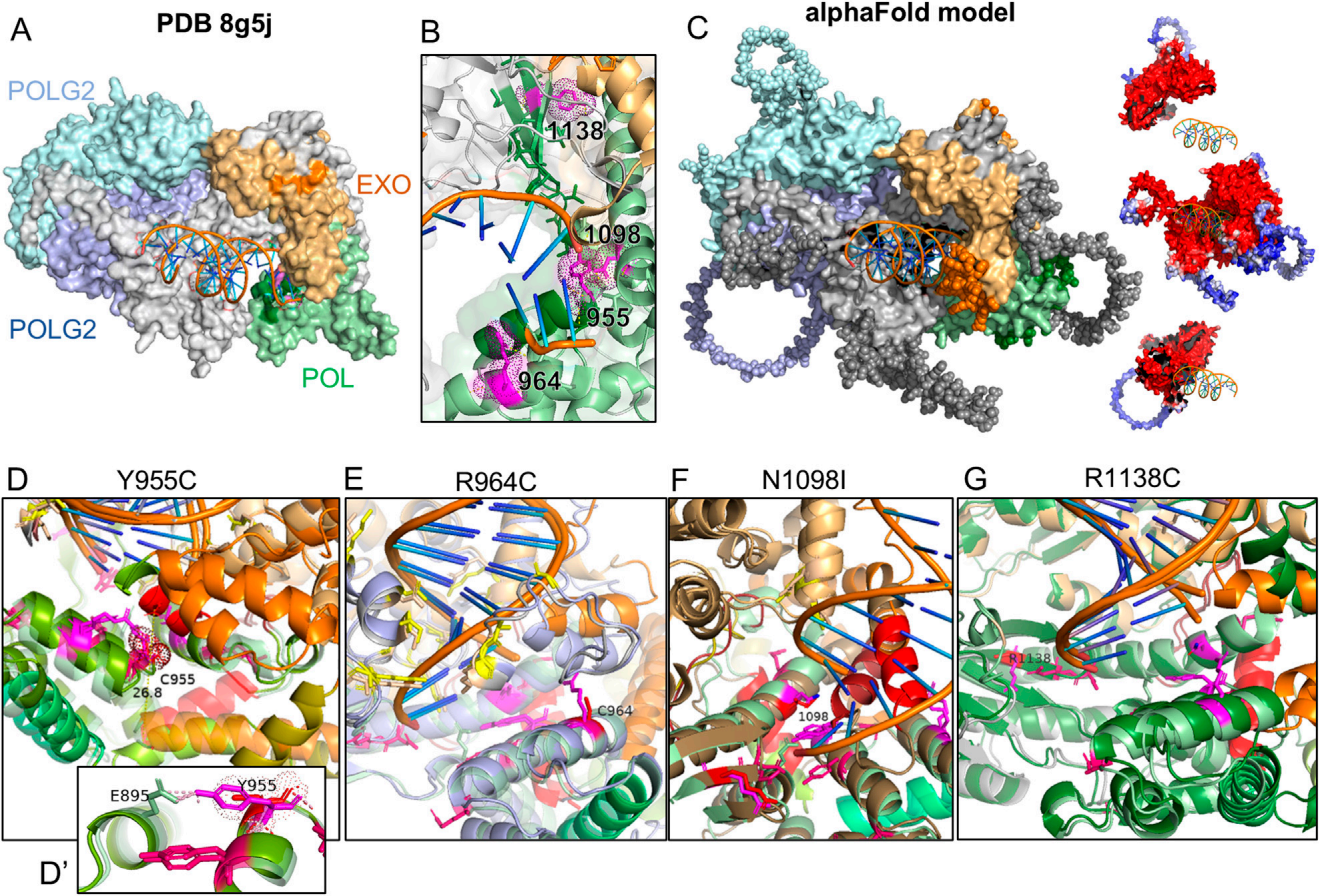
AlphaFold modeling of POLG variants and impact on the POLG/POLG2/dsDNA complex. **(A)** Crystal structure PDB8g5j of the human POLG holoenzyme complexed with DNA. **(B)** Zoomed view of the catalytic domain and residues of interest (magenta). Blues: POLG2s, gray: POLG, orange: POLG exonuclease domain, green: polymerase domain. **(C)** AlphaFold-predicted structure of human POLG (NP_001119603) complexed with two POLG2 subunits (NP_009146) and a short sequence of DNA. Residues shown as spheres were presumably too mobile to be resolved in the crystal structure. Insets of POLG and POLG2s illustrate model confidence (blue low; red high). **(D–G)** Catalytic domains modeled with POLG variants. Wild-type is shown as partially transparent. WT helices between Q976 and L1083 are colored green-olive–orange–red-brick. Residues associated with PEO are shown in hot pink. The variant in each panel is shown in red, with the WT residue in magenta.

To gauge the 3-dimensional location of the investigated variants relative to known pathogenic variants, we illustrated their side chains on the crystal structure of human POLG bound to DNA (PDB: 8g5j) using PyMOL (Figures 1A, B). R1138 is the most distal from the catalytic site of the polymerase domain, while Y955, R964, and N1098 are oriented toward the nascent DNA strand. To model potential changes in the morphology of the POLG:DNA interaction, we used AlphaFold Server to predict the structure of double-stranded DNA (sequence from 8g5j) in a trimeric complex with POLG and two POLG2 subunits. The predicted structure closely matched the published crystal structure with expectedly lower certainty for more mobile portions that were not resolved in the crystal structure (Figure 1C). Y955C removes a polar contact with E895 of the neighboring helix (Figure 1D), accompanied by a predicted remodeling of polymerase domain helices and a ∼28 Å shift of the 1,056–1,066 helix (Figure 1D, opaque red vs. transparent red for WT) into the catalytic site. The R964C variant showed little obvious structural change, where DNA-interacting residues (shown in yellow) appear to maintain their relationship with the DNA strand (Figure 1E). In three of five AlphaFold models, substitution of the polar N1098 with hydrophobic I1098 caused the 1,056–1,066 helix to move from the exterior aspect of the polymerase domain into the active site of the polymerase domain (Figure 1F, opaque red helix vs. transparent). By comparison, when we modeled the disease-associated N1098K, the top two models showed little change in the morphology of the catalytic site, lateral displacement, or disruption of the 328–336 helix in two models (shown in orange in Figures 1E, G) along with shallower seating of the DNA strand, and obstruction of the active site by the 1,056–1,065 helix in the fifth model (Supplementary Figure 1). AlphaFold showed modest variations in the low-certainty, lateral aspect of the polymerase domain for the R1138C variants but did not predict clear changes within the catalytic site (Figure 1G).

### 3.2 High content imaging of mtDNA and ploidy in cultured cells

**FIGURE 2:**
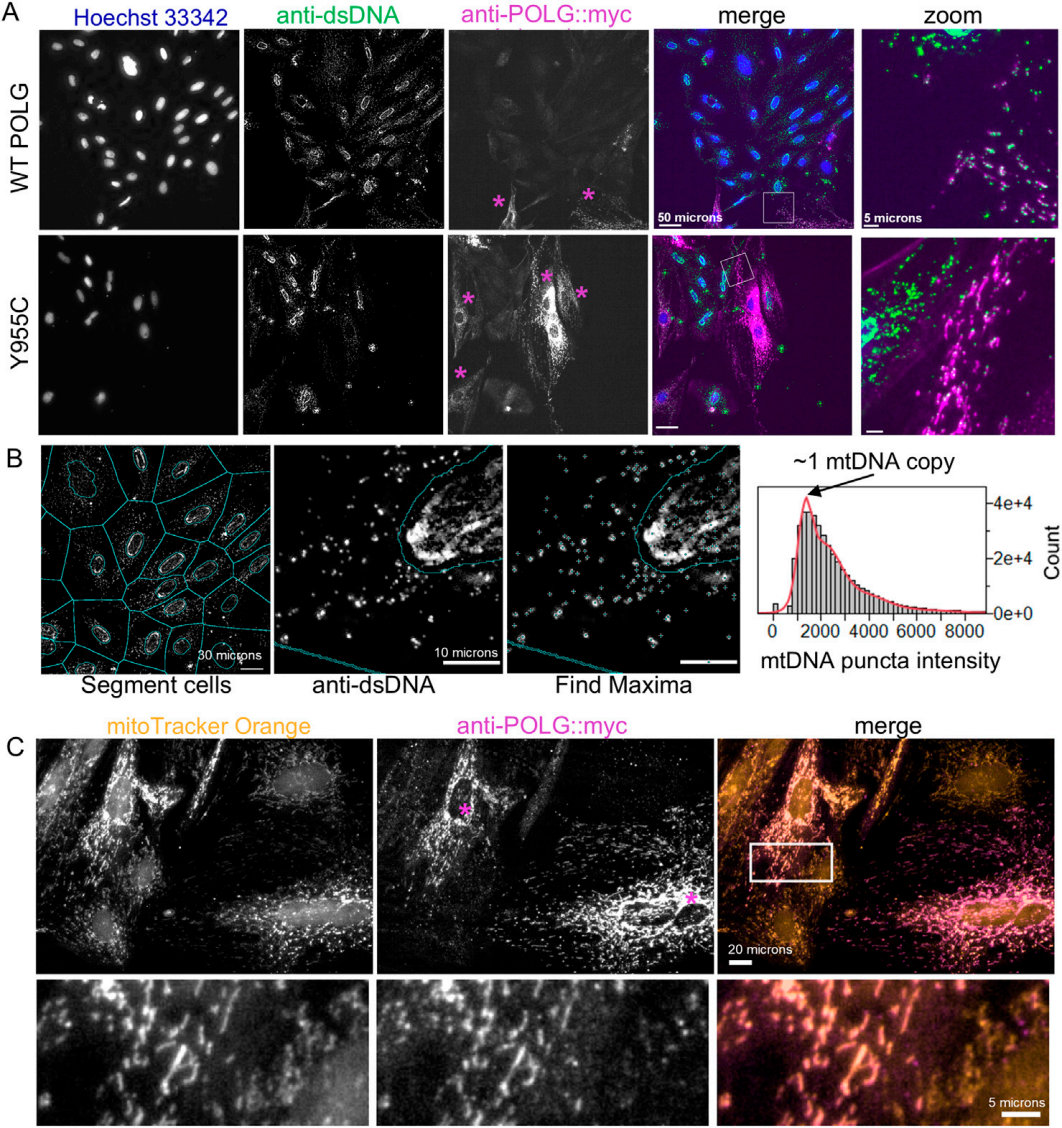
Image-based estimation of mitochondrial DNA copy number and localization of exogenous POLG. **(A)** A7r5 cells transfected with wild-type or Y955C POLG variants and then fixed and labeled with anti-dsDNA and anti-Myc (i.e., Myc-tagged POLG) and Hoechst-33342. “Zoom” panels are enlarged regions in box in “merge” panels. **(B)** Automatically generated cellular ROIs are generated by dilating Hoechst-defined nuclear ROIs by 4 pixels and then expanding to the edges of the Voronoi tessellation boarders with neighboring cells. dsDNA puncta are identified as local maxima and assigned to the cellular ROIs that exclude the nucleus. For each set of staining, the distribution of puncta intensities is fitted to a 10x Gaussian model, assuming that the most abundant puncta represent nucleoids with a single mtDNA copy. mCN is estimated as the puncta intensity divided by this quantal intensity. **(C)** A7r5 cells were stained with MitoTracker Orange (50 nM) and then fixed and labeled with the anti-Myc antibody to confirm that exogenous POLG is localized to the mitochondria, as expected. In **(A, C),** purple asterisks indicate cells that are immune-reactive for Myc (POLG).

We have previously demonstrated that the A7r5 rat aorta smooth muscle cell line is a useful culture model to assess calcium signaling and phenotypic switching (Kim et al., 2021). Here, we use this model to study the effects of POLG loss-of-function variants on smooth muscle phenotype and metabolism. For end-point analyses of cell proliferation, mtDNA content, and transfection efficiency, we plated A7r5 cells on 12-mm glass coverslips or Greiner µclear 96-well plates (Greiner 655-090) and transfected them with constructs expressing Myc and FLAG^®^-tagged human POLG or variants created by site-directed mutagenesis. Following the post-transfection growth period, we fixed cells and immunestained them for doubled-stranded DNA (dsDNA) and the Myc or FLAG-tag on the exogenous POLG and then stained them with Hoechst-33342 to label nuclei and assess nuclear DNA content. Notably, Hoechst-33342 was not visible within mitochondria. Figure 2A shows images for the wild-type (WT) POLG and the Y955C variant. Zoomed regions illustrate dsDNA in Myc-labeled mitochondria-shaped organelles in transfected cells (Figure 2A). Myc staining of exogenous POLG correctly localized to polarized mitochondria labeled with MitoTracker Orange CMTMRos (Figure 2C). We estimated cellular levels of mtDNA by quantifying the summed intensity of dsDNA puncta per cell in Voronoi ROIs that exclude the Hoechst-defined nuclear ROIs, as previously validated (Figure 2B) (Li et al., 2018). Briefly, the distribution of puncta intensities was fitted to a 10x Gaussian distribution to estimate the mean quantal nucleoid intensity for a given experiment, and then the sum of puncta intensities per cell was divided by that value (Figure 2B).

### 3.3 POLG variants induce a mitogenic effect that is proportional to the loss of mtDNA in end-point analyses

**FIGURE 3:**
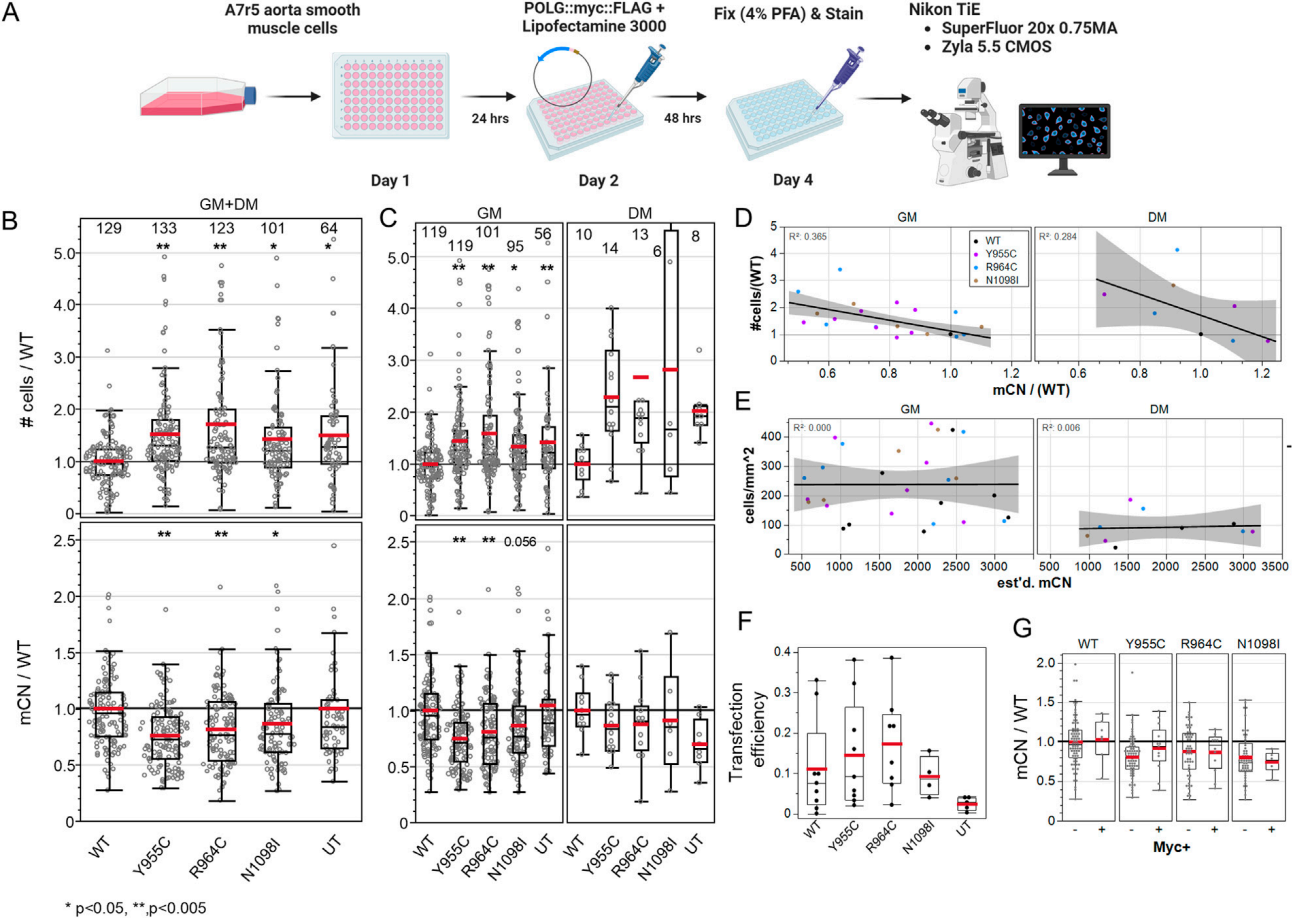
A7r5 cell growth and mitochondrial DNA copy number are affected by POLG variants. **(A)** Illustrated experimental outline. **(B)** Summary of all A7r5 cell density and mCN measures per field of view normalized to the mean value of wild-type fields of view per plate for each media. Numbers above columns show the number of fields of view analyzed. *p < 0.05 and **p < 0.005 for Dunnett’s *post hoc* tests *versus* wild-type. UT–untransfected. **(C)** Data in **(B)** separated by media condition. **(D)** Linear regression of plate-wise means of the normalized cell density as a function of normalized mCN with *R* ^2^ correlation coefficients in upper left corners. GM and DM indicate media conditions. **(E)** Linear regression of the absolute cell density and estimated mCN. **(F)** Fractions of cells positive for Myc immunoreactivity. **(G)** Normalized mCN per FOV when split between Myc-negative and Myc-positive cells. # Cells analyzed: 25,733 in GM, 1,461 in DM. FOVs: 489 in GM, 49 in DM. Plates: 7 GM, 2 DM.

We hypothesized that over-expression of loss-of-function POLG variants would reduce mCN in transfected cells and influence their cell-cycle activation. Figure 3A shows the experimental scheme. We normalized the cell density to the mean of WT POLG-transfected wells on each plate for each media condition. We normalized mCN in a similar fashion to minimize the plate-wise variance in mCN. Staining for cytosolic dsDNA puncta in cells in transfected cultures (combining GM and DM-treated cells) was reduced by 27% by Y955C, 20% by R964C, and 14% by N1098I over-expression (Figure 3B), while untransfected cultures showed mCN that was equivalent to WT-transfected cultures. In these same cultures, the cell density normalized to the density of WT-transfected cells on a per-experiment basis was increased by 42%–75%. Notably, untransfected cultures showed higher density than cultures transfected with WT POLG, as expected due to the toxicity and stress associated with transfection. The differences were persistent when the dataset was separated for cells grown in GM or DM (Figure 3C). While the number of experiments performed in DM was insufficient to reach significance, the trends in mCN and cell density closely matched those seen in GM.

The normalized cell density correlated well with relative changes in the loss of mtDNA immunolabeling across the POLG variants at plate-level means (*R* ^2^ = 0.37 GM, 0.28 DM) (Figure 3D). To assess the possibility that reduction in mCN was caused by cells being small in more dense cultures, we plotted absolute cell density against our estimates of absolute mCN and found that there was no correlation (*R* ^2^ = 0.00–0.01), suggesting that mCN was not simply a function of cell density (Figure 3E). This was even more evident in the single field-of-view plot for each plate, where plates with a higher cell density did not show lower mCN (Supplementary Figure 2A). We further examined whether POLG-associated changes in mCN might be better correlated with the mean Voronoi ROI size (i.e., smaller in more dense cultures), but here, the correlation was very poor (*R* ^2^ = 0.05 GM, 0.08 DM) (Supplementary Figure 2B). Finally, we examined the MitoTracker Orange staining as a measure of the total mitochondrial mass per cell, as a correlate of mCN. We employed ImageJ 32-bit thresholding that does not count sub-threshold pixels toward the total mitochondria area per cell and observed a poor correlation in GM-treated cells (*R* ^2^ = 0.09) but a good correlation in a smaller sample of DM-treated cells (*R* ^2^ = 0.65).

A surprising observation was the level of changes observed in mCN given the transfection efficiency (i.e., cells staining positive for the POLG-Myc tag, ∼10–20%) (Figure 3F). While we had expected extensive loss of mCN in transfected cells, we observed that the decrease in mCN staining was similar in Myc-negative and Myc-positive cells in the same wells (Figure 3G). This suggested that some form of diffusible factor could be impacting cell growth and dsDNA immunolabeling.

### 3.4 POLG variants did not induce signs of polyploidy or endoreduplication

**FIGURE 4:**
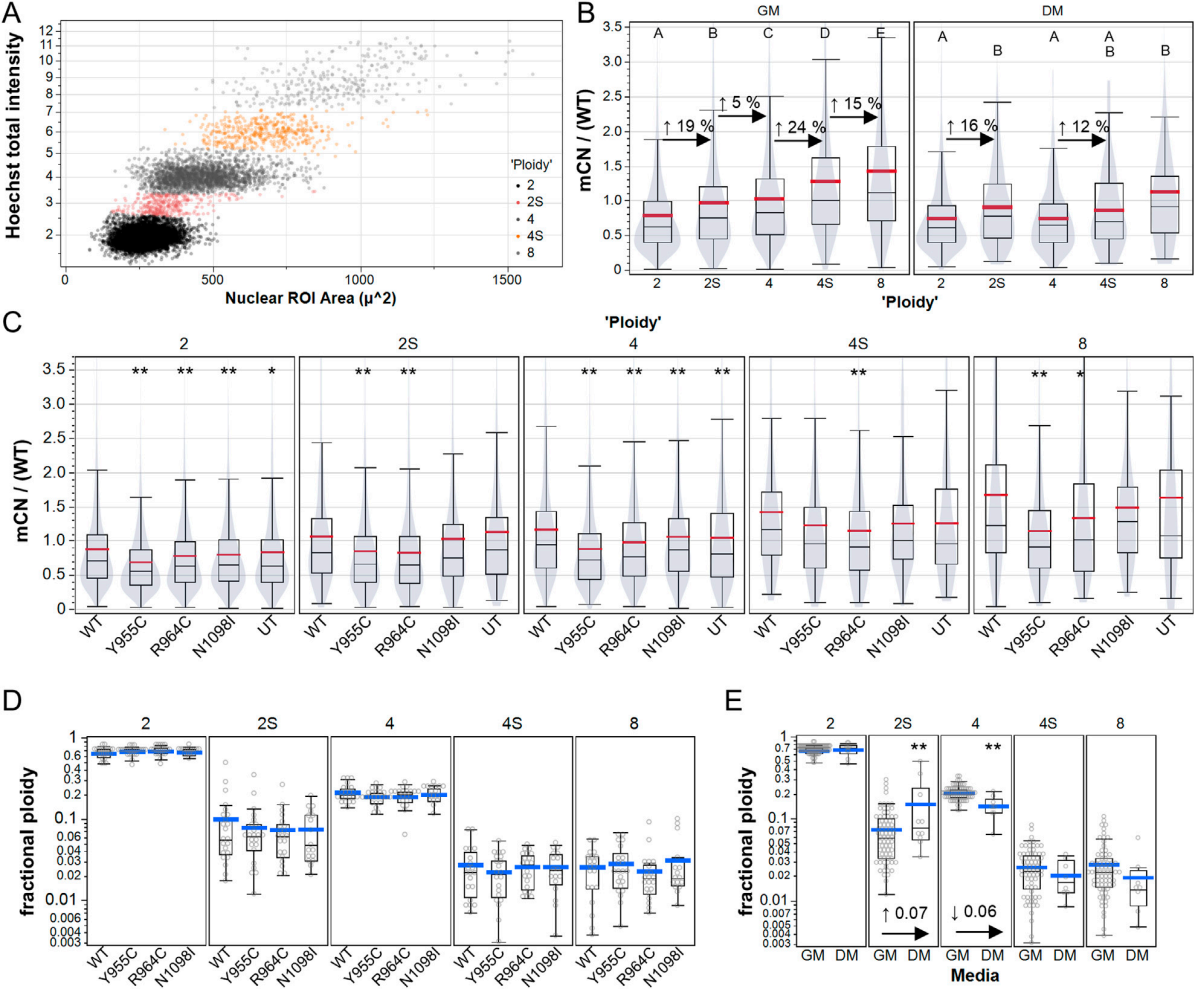
High content analysis of A7r5 mCN over cell cycle and polyploidy. **(A)** Example of manual gating of integrated Hoechst-33342 intensity and nuclear area to label A7r5 cells as follows: 2: diploid G_0_/G_1_, 2S: diploid S-phase, 4: diploid G_2_/M or tetraploid G_0_/G_1_, 4S: tetraploid S-phase, and 8: tetraploid G_2_/M or octoploid G_0_/G_1_. **(B)** Mitochondrial DNA copy number (mCN/WT) shown for all transfection conditions over gated cell cycle and ploidy groups. For each media condition, means that share a letter above the dataset are not different (*p* < 0.05) by Tukey’s honest significant different test. **(C)** Impact of POLG variants on mCN in each ploidy group, where ** indicates *p* < 0.005 for Dunnett’s *post hoc* tests *versus* wild-type. In B and C, violin plots indicate that data are summarized at the single-cell level. **(D, E)** Fraction of cells per field of view in each ploidy category for **(D)** each POLG variant and for **(E)** all POLG variants separated by media conditions. ** indicates *p* < 0.005 in comparison of DM vs. GM for each category. # Cells analyzed: 25,733 in GM, 1,461 in DM. FOVs: 489 in GM, 49 in DM. Plates: 7 GM, 2 DM.

Cellular depletion of mtDNA to create Rho0 cells is reported to induce mitotic abnormalities and accumulation of aneuploid cells and cell-cycle dysregulation (Donthamsetty et al., 2014), but whether this occurs in cells with modest reductions in mCN is not clear. We plotted total nuclear Hoechst intensity *versus* nuclear size (area) to gate cellular ploidy levels, as previously described (Figure 4A) (Kim et al., 2021). Consistent with the notion that mtDNA replicates in preparation for cell division, we observed increases in the mtDNA immunoreactivity (∼20%) in cells gated as 2N and 4N cells in the S-phase (Figure 4B). Although 4N and 8N cells had increased mtDNA labeling compared to 2N cells, the increase was much less than 2-fold and 4-fold relative to 2N cells, suggesting that polyploid cells have relatively lower mCN per nuclear genome equivalent. In this experiment, we measured the changes in mCN caused by the Y955C, R964C, and N1098I variants and observed reduced mCN (on a cell-wise basis) in all gated populations, with less clear differences in the less numerous 2N and 4N S-phase cells (Figure 4C). In contrast, no difference was evident in the fraction of cells in any gated population as a function of the transfection of POLG variants (Figure 4D), whereas cells grown in differentiation media exhibited the expected increase in the fraction of cells in the S-phase and decreased fraction of cells in the G2 and M phases, as observed by the reduction in “4N” cells (Figure 4E).

### 3.5 POLG variants’ impact on the absolute mtDNA copy number

**FIGURE 5:**
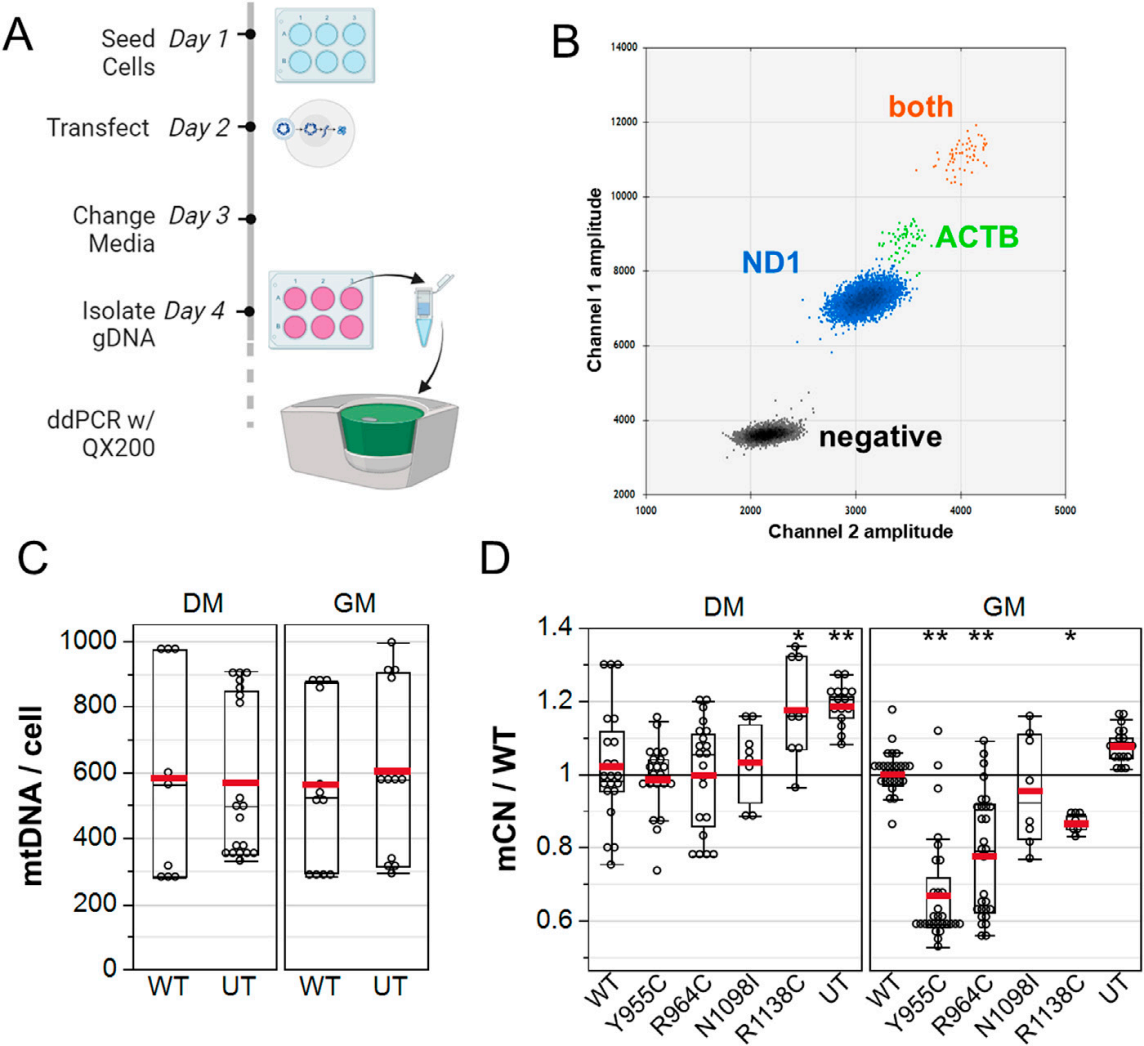
ddPCR analysis of A7r5 mitochondrial DNA copy number. **(A)** Illustration of the experimental protocol. **(B)** 2D droplet intensity plot of EvaGreen supermix duplex assay. Gray: –droplets containing no template. Blue: droplets with only mitochondrial DNA detected with ND1 primers. Green: droplets with only nuclear DNA detected with ACTB primers. Orange: droplets containing both mitochondrial and nuclear DNA. **(C)** Summary of absolute mitochondrial DNA copy number per cell for A7r5 grown in GM *versus* DM. **(D)** mCN normalized to mean WT results for each transfection condition in each media. UT is untransfected. *p < 0.05 and **p < 0.005 for Dunnett’s *post hoc* tests *versus* wild-type. Data show technical quadruplicates of seven independent experiments (two included N1098I and R1138C).

To further validate whether the POLG variants reduced mCN, we quantified mCN by droplet digital PCR at 48 h post transfection (Figure 5A) using primers that were designed to selectively amplify rat mitochondrial ND1 and primers that target a nuclear site immediately 5′ of the ACTB gene that we previously reported to amplify three loci in the rat genome (Li et al., 2018). A 2D plot of the intensity of EvaGreen-labeled droplets revealed four distinct droplet populations: droplets with no template, with only ND1-containing DNA, with ACTB only, and with both ND1 and ACTB templates (Figure 5B). We performed a two-way ANOVA on a subset of our data, in which GM- and DM-treated cultures were isolated in parallel to assess whether WT POLG overexpression affected the absolute number of copies of mtDNA per cell. DM-treated (576 ± 53) and GM-treated (584 ± 54) cultures had equivalent absolute mCN. Similarly, mCN in cultures transfected with WT POLG (574 ± 58) was equivalent to that in untransfected cultures (587 ± 48), which were measured in technical quadruplicates from three independent experiments (Figure 5C). Notably, between-experiment variance (coefficient of variance 0.37) was greater than within-experiment variance (average CV 0.20). To assess whether POLG variants affected mCN, we normalized mCN to the WT transected values for each media condition for each experiment. As with imaging experiments, Y955 and R964C reduced mCN in GM-treated cultures by 28% ± 3% and 19% ± 3%, respectively, as assessed by one-way ANOVA, followed by a Dunnett’s test against WT as the control level. Although N1098I did not reduce mCN in either of the media, Y1138C reduced mCN in GM. In DM-treated cultures, both Y1138C and untransfected cells showed 18%–20% increases in mCN (Figure 5D).

### 3.6 POLG variants minimally affect ΔΨm

**FIGURE 6:**
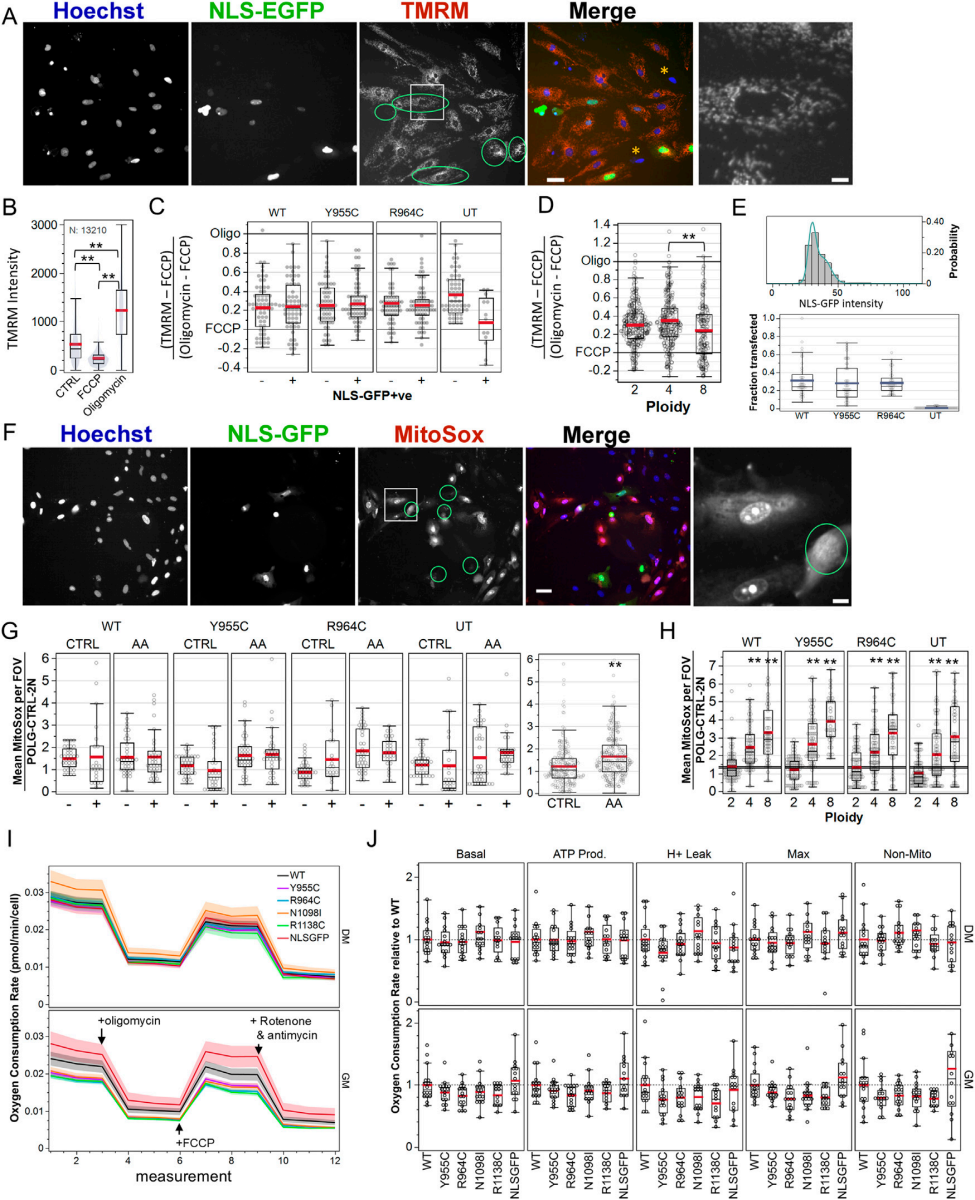
Loss-of-function POLG variants minimally affect mitochondrial membrane potential, ROS production, and cellular respiration in A7r5 cells. **(A)** Example image of A7r5 cells transfected with pPOLG:P2AT2A:EGFP3xNLS labeled with Hoechst 33342 and TMRM (25 nM) and treated with oligomycin (10 µM). The green circle overlays highlight transfected cells, and the white box is zoomed in on the far right. Orange asterisks highlight cells with minimal TMRM staining. Scale bars: 50 µm for composite images and 10 µm for zoomed images. **(B)** Average TMRM intensity for all 13,210 cells in one of two experimental replicates. **p < 0.005 for Tukey’s honest significant different test. **(C, D)** TMRM values in the control-treated well, where each cell was normalized as its TMRM intensity less the mean FCCP value and divided by the difference between the mean of oligomycin- and FCCP-treated wells. Data are compiled from four fields of view in each quadruplicate well in each of two independent experiments. **(E)** Example distribution of nuclear GFP mean intensity and triple Gaussian fit. Cells with mean intensity higher than mean + (3 x SD) of first Gaussian peak are defined as transfected. Summary statistics of the transfection efficiency are shown below. **(F)** Representative images of MitoSOX (5 µM) in cultured transfected with WT pPOLG:P2AT2A:EGFP3xNLS treated with antimycin A (1 µM). Green circle overlays represent transfected cells, and the white box is zoomed in on the far right. Scale bars: 50 µm for composite images and 10 µm for zoomed images. **(G)** MitoSOX intensity was measured using nuclear regions of interest and normalized to the means of control-treated, WT-transfected cells on each of two independent experiments, imaging four fields of view in each quadruplicate well. AA indicates wells treated with antimycin A (1 µM) to stimulate ROS production. **p < 0.005 for two-sample *t*-test. **(H)** Data in **(G)** are re-plotted by simplified cell ploidy. ** indicates *p* < 0.005 for Dunnett’s *post hoc* tests *versus* 2N. **(I)** A7r5 oxygen consumption rates per cell in Seahorse XFe Mito Stress Test. Lines show mean plus standard error bands for two independent experiments with 6–8 technical replicates each. Oligomycin (1 µM), FCCP (1 µM), rotenone, and antimycin A (1 µM) were added, as indicated with the arrows. **(J)** Oxygen consumption rate normalized to POLG separately for GM and DM conditions. One outlier ( > 3) in the GM for non-mitochondrial OCR for NLSGFP (4.283) and one well corresponding to five points in N1098I DM were removed for clarity but not removed from statistical analysis. For each experimental paradigm (TMRM, MitoSOX, and Seahorse), data show the results of two independent experiments, with technical quadruplicate wells for TMRM and MitoSOX and eight technical replicates per Seahorse plate.

We assessed mitochondrial membrane potential microscopically by labeling cells transfected with the pPOLG-P2AT2A-EGFP3xNLS variants with TMRM, where nuclear GFP facilitated identification of transfected cells without the need for *post hoc* anti-Myc/FLAG immune-labeling, where ROIs were generated and seeded again around Hoechst-stained nuclei (Figure 6A). We performed three independent experiments with technical quadruplicate wells. Visual inspection of images suggested that NLS-GFP-positive cells did not exhibit obvious mitochondrial depolarization (Figure 6A). We fit the distribution of nuclear EGFP intensity in untransfected cells with a triple Gaussian distribution and used the mean plus three standard deviations of primary distribution as the threshold to label transfected cells (Figure 6E). This indicated an average transfection efficiency of ∼25% (Figure 6E bottom panel).

Separate wells contained untreated cells and cells treated with FCCP (0.3 µM) to depolarize mitochondria and oligomycin (10 µM) to inhibit the F1F0-ATP synthase and hyperpolarize ΔΨ_m_ (Figure 6B). We then normalized TMRM 32-bit thresholded intensity in control wells to the mean value of wells treated with FCCP and oligomycin for each of three independent experiments to account for plate-wise differences in the TMRM intensity (Figure 6C). We restricted the analysis to cells that were gated for ploidy 2N–8N and summarized the normalized TMRM values by field of view (FOV) and performed a two-way ANOVA with POLG variants and NLS-GFP expression (i.e., + or –) as random-effects variables. The POLG variant was not a significant factor (*p* = 0.76), with least square mean ± standard error of WT 0.23 ± 0.02, Y955C 0.26 ± 0.02, R964C 0.27 ± 0.02, and untransfected 0.22 ± 0.04. Retrospective power analysis indicated that a difference of 0.04 would be 50% likely to be detected, suggesting that the absence of differences was unlikely due to the underpowered experiment. There was a significant interaction effect between the POLG variant and NLS-GFP labeling (*p* < 0.001). Linear contrasts failed to detect differences between NLS-GFP + or – cells for POLG, Y955C, or R964C (*p* > 0.4 for each). Instead, we found a difference between untransfected cells (NLS-GFP negative vs. the small number of false-positives). Thus, POLG variants did not affect ΔΨ_m._

We made two notable ancillary observations. First, approximately 50% of the cells in most fields of view in transfected wells (∼30% in untransfected wells) showed less TMRM staining that was unrelated to nuclear size (i.e., not coincident with apoptotic nuclear condensation) and not associated with Hoechst staining intensity (i.e., probably not linked to cell cycle). These are indicated by orange asterisks in Figure 6A. Second, TMRM staining intensity in 4N cells was marginally (but not statistically) higher than that in 2N cells, but significantly higher than that in 8N cells (Figure 6D). The 4N population includes both genuinely tetraploid cells and diploid cells in the G2/M phase, with the latter being a majority of this population and presumably accounting for this elevation.

### 3.7 POLG variants do not increase ROS production

Mitochondrial dysfunction is often assumed to result in excess production of radical oxygen species (ROS). We assessed ROS production microscopically by labeling cells transfected with the pPOLG-P2AT2A-EGFP3xNLS variants with MitoSOX and Hoechst labeling (Figure 6F). We performed two independent experiments with technical quadruplicate wells. As a positive control to stimulate ROS production, an equal number of wells served as the control or were treated with 20 µM antimycin A (AA) to inhibit complex III. Again, results were summarized at the level of cell means per FOV, where FOV values were normalized to the per plate mean of the WT-transfected, control-treated, 2N cells. Due to the high concentration of MitoSOX used (5 µM), the majority of the signal was localized to the cell nucleus. We performed a three-factor ANOVA for variant, NLS-GFP positivity, and drug treatment (control vs. AA). Variant (*p* = 0.39) and NLS-GFP positivity (*p* = 0.37) did not affect MitoSOX intensity. Drug treatment significantly affected ROS (*p* < 0.0001), with control values at 1.22 ± 0.06 and antimycin A at 1.66 ± 0.05 (Figure 6G right panel). A significant interaction between drug treatment and variant (*p* = 0.038) was largely due to the significant difference between MitoSOX signal in control-treated and antimycin A-treated Y955C- and R964C-transfected groups (Tukey’s honest significant difference test, *p* < 0.05).

As with TMRM, MitoSOX labeling was negligible in some cells and high in others (examples in Figure 6F). In a three-factor ANOVA drug treatment (*p* < 0.0001), NLS-GFP positivity (*p* = 0.004), drug x plasmid (*p* = 0.001), and drug x NLS positivity (0.0002) were significant factors or interactions in the fraction of cells gated as “high” for MitoSOX (i.e., above background intensity). “Plasmid” was a marginal factor (*p* = 0.059) [data not shown]. Control-treated cells were 50.2% ± 2.1% high vs 62.2% ± 2.0% in AA-treated cells, while NLS-GFP-positive cells were 59.5% ± 2.0% high vs 51% ± 2.2% for GFP-negative cells. Notably, WT-transfected cultures (61.0% ± 3.0% high) had a larger fraction of cells with high MitoSOX labelling than Y955C (52.7% ± 2.7% high), R964C (57.3% ± 2.9%), or untransfected (50.6% ± 3.0%) cultures. Overall, this analysis indicated that over-expressing Y955C or R964C did not result in elevated ROS generation, specifically in the transfected cells or in their neighboring cells.

However, we did observe that elevated mean MitoSOX intensities (in nuclear ROIs) were pronounced in 4N and 8N cells relative to 2N cells (Figure 6H). Here, cell means were additionally summarized by ploidy level. Neither plasmid (*p* = 0.29) nor plasmid–ploidy (*p* = 0.24) were significant factors in a two-way ANOVA, while ploidy (*p* < 0.0001) was a significant factor for MitoSOX intensity. Normalized MitoSOX intensities for 2N, 4N, and 8N were 0.95 ± 0.08, 2.05 ± 0.08, and 2.64 ± 0.10, respectively, in control-treated cells. This illustrated the assay’s ability to detect differences in cellular ROS production.

### 3.8 POLG variants do not impair population-level cellular respiration

The Seahorse XF Cell Mito Stress Test allowed us to explore the effect of the POLG variants on key parameters of mitochondrial metabolism in A7r5 VSMCs by sequentially inhibiting the ATP synthase activity, uncoupling respiration, and then inhibiting complexes I and III to measure basal respiration, ATP-linked respiration, proton leak, maximal respiration, and non-mitochondrial oxygen consumption (Figure 6I). We normalized these metrics for cell density, counting *post hoc* Hoechst and NLS-GFP labeled nuclei using the Nikon TiE. The average cellular density was greater in GM conditions compared to DM conditions, with the transfection efficiency remaining consistent with those of previous experiments, at approximately 20% (Supplementary Figure 4A, C). Metabolic measurements were stable over a wide range of cellular plating densities (Supplementary Figure 4B). Additionally, baseline OCR, when normalized per cell, was comparable between plasmids and showed a slight increase in DM conditions (Supplementary Figure 4C). This may indicate a shift toward a more oxidative phenotype in the “low” (5 mM) glucose media. To further compare between transfection conditions, we normalized OCR measures to the WT mean per plate for each media condition and compared the OCR as a function of transfected plasmids by one-way ANOVA for each category of respiration by media (Figure 6J, six outliers > 2.3 for NLS-GFP GM and N1098I DM are not shown). While mean values for POLG variants tended to average ∼10–20% below the WT-transfected conditions, for those groups with an ANOVA *p*-value <0.05 (GM: basal OCR *p* = 0.026, ATP production *p* = 0.02, max. respiration *p* = 0.002, and non-mitochondrial resp. *p* = 0.04), *post hoc* Dunnett’s tests against WT failed to show any differences between WT and the POLG variants.

To further assess the collective effect of the POLG variants, we compared WT OCR values to those of the variants for each media condition (Supplementary Figure 4D). In the GM media condition, all OCR categories were significantly lower than those of the WT-transfected cultures (GM: basal OCR *p* = 0.022, ATP production *p* = 0.042, H+ leak *p* = 0.008, max. respiration *p* = 0.006, and non-mitochondrial resp. *p* = 0.007), with mean decreases of 12%–21% (% decrease relative to WT: basal: 14% ± 3%, ATP production 12% ± 2%, proton leak 21% ± 4%, max. 19% ± 3%, and non-mitochondrial 19% ± 3%). For the DM condition, the grouped variants were not different from WT (ANOVA *p*-values: 0.51–0.94), with mean differences of +3% to −8%. The impact of media treatment was consistent with more extensive reduction in mCN in the GM.

### 3.9 A diffusible factor mediates the mitogenic effect of loss-of-function POLG variants, evidenced by live-cell imaging

**FIGURE 7:**
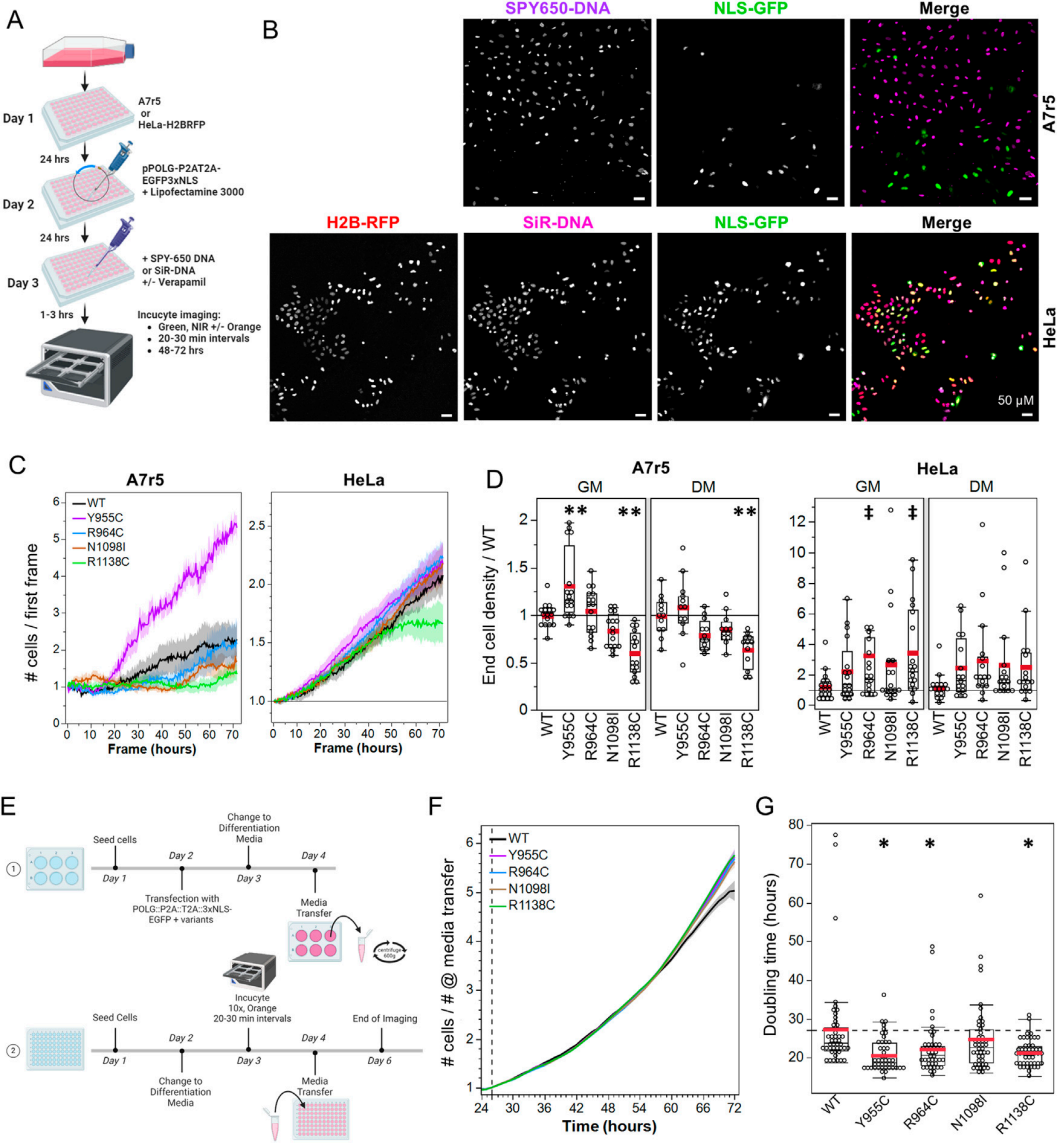
Loss-of-function POLG variants mediate a mitogenic effect *via* diffusible messenger. **(A)** Protocol illustration. **(B)** IncuCyte exemplar images of pPOLG:P2AT2A:EGFP3xNLS-expressing A7r5 (top) and HeLa (bottom) nuclei labeled with SPY650 DNA (3000x dilution) and SiR-DNA (0.5 µM), respectively. HeLa stably express H2B-RFP. **(C)** Example growth curves normalized to the number of cells in the first frame, with mean banded by standard error for four fields of view. A7r5 are in DM, and HeLa cells are in GM. **(D)** Effect of POLG variants on final cell density, normalized to the density in WT-transfected cells. **p < 0.005 for Dunnett’s *post hoc* tests *versus* WT, ‡*p* < 0.05 for Dunnett’s *post hoc* test vs. WT for combined GM + DM data. Data show two fields of view in duplicate wells on each of three independent experiments. **(E)** Illustration of the media transfer protocol. **(F)** Growth curve of naïve HeLa cells treated with conditioned media from transfected HeLa cells, which were normalized at 26 h when the media was added. **(G)** Doubling time in hours for WT POLG and variants. *p < 0.05 for Dunnett’s *post hoc* tests *versus* WT. Data show the summary of single fields of view imaged in eight duplicate wells (i.e. eight wells per plasmid) on each of three independent experiments.

With the goal of assessing changes in population doubling times and the persistence of transfected cells in the population over time, we employed multi-day live-cell imaging using the IncuCyte system. To monitor cell growth over time, we aimed to image a fluorescent nuclear marker dye. Far-red nuclear stains are claimed to be less cytotoxic than ultraviolet-excited dyes such as Hoechst (Purschke et al., 2010; Lukinavičius et al., 2015; Sen et al., 2018), so we employed SiR-DNA and Spy650-DNA (Spirochrome) (Figures 7A, B). In our initial studies at 0.25 µM, SiR-DNA had little impact on 48-h HeLa cell growth whether imaged or not, where cell growth was monitored *via* a stably expressed H2B-RFP. At 0.5 µM, SiR-DNA did not impact growth when not imaged, but it slightly reduced growth when imaged. A7r5 cell growth (48 h) was comparable whether 0.25 or 0.5 µM SiR-DNA was present throughout the 48 h time point and whether imaged or not (Supplementary Figure 5). For these experiments, we opted for nuclear segmentation with StarDist, which performed better than our threshold-based segmentation in densely grown HeLa cells and with variable levels of H2B-RFP within a field of view (not shown).

We transfected A7r5 and HeLa cells in 96-well plates, removed transfection reagents 24 h later, and allowed 24-h recovery in GM or DM before imaging the cell growth for 72 h (Figure 7A, example curves are in Figure 7C). HeLa cells grew well following transfection, but the stress of long-term imaging and transfection led to poor growth in A7r5 cells. Since A7r5 growth was not exponential in these conditions, we compared the relative growth in each field of view (final # cells/# cells in first frame) normalized to the WT-transfected cells for each media and performed separate one-way ANOVAs comparing the cell growth between pPOLG-P2AT2A-EGFP3xNLS variants for each cell type and media condition with *post hoc* Dunnett’s tests. In A7r5 cells growing in GM (ANOVA *p* < 0.0001), Y955C increased cell growth (*p* = 0.002), while R1138C reduced growth (*p* < 0.0001), while in DM (ANOVA *p* < 0.0001), R1138C reduced growth (*p* = 0.0005) (Figure 7D). HeLa growth in GM and DM was not affected by the plasmids (ANOVA *p* = 0.16 and 0.22, respectively). Given consistent trends for increased growth in HeLa cells in both media conditions, we performed an ANOVA for the combined GM + DM dataset (*p* = 0.021), with R964C (*p* = 0.011) and R1138C (*p* = 0.017) showing significantly increased cell density.

The earlier observations of reduction in mtDNA staining and reduced mCN throughout transfected cultures suggested that the mitogenic effects of the POLG variants were mediated by a diffusible factor. To test this possibility, we treated naïve cells that were growing the IncuCyte for 24 h in low serum media (HeLa DM) with conditioned media from HeLa cells transfected with the variants (protocol outline in Figure 7E). We normalized HeLa growth curves to the cell density at the point of addition of conditioned media (Figure 7F). These growth curves exhibited exponential growth, allowing calculation of doubling times. A one-way ANOVA by plasmid was significant (*p* = 0.0002), with doubling times being lower for cells treated with conditioned media from Y955C (20.5 ± 1.1 h, *p* = 0.0003)-, R964C (22.1 ± 1.2 h, *p* = 0.009)-, and R1138C (21.2 ± 1.1 h, *p* = 0.001)-transfected cells *versus* WT-transfected cells (27.3 ± 1.2 h) (Figure 7G). This presented encouraging evidence of a diffusible mitogenic factor being released from cells over-expressing the LOF POLG variants, but not WT POLG.

### 3.10 NF-Kß and mitochondrial ROS likely contribute to the mitogenic effects of POLG variants

**FIGURE 8:**
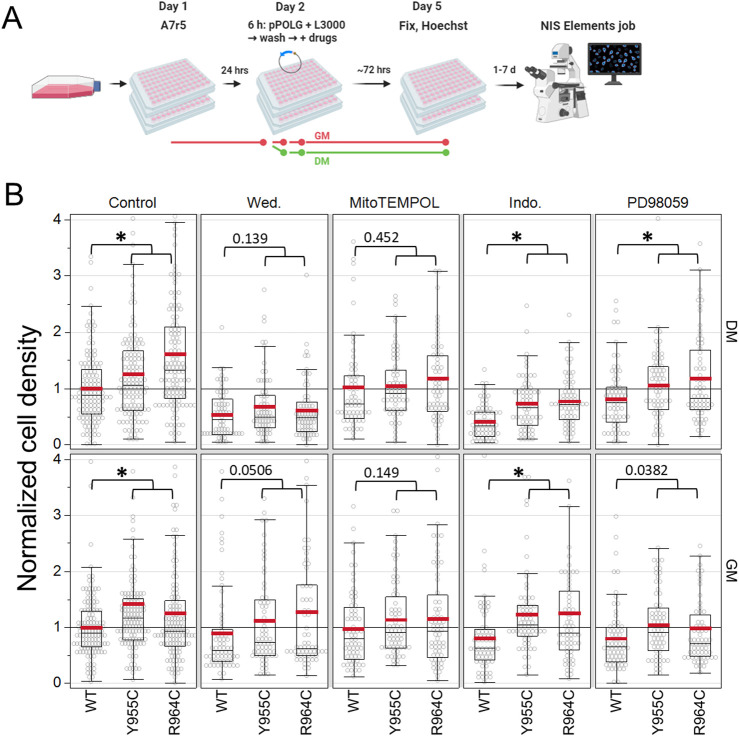
Pharmacological characterization of mitogenic signaling in A7r5 cells. **(A)** Experimental illustration. **(B)** Summary statistics for cell growth (# cells/field of view) normalized to control-treated (no treatment), WT-transfected wells per plate by media condition. Four independent experiments per media condition, 128 fields of view for control, and 64 fields of view for drug treatments, with a range of 795–35,196 cells analyzed per condition. *p < 0.05 for Dunnett’s test *versus* WT for each drug treatment/media combination. Wed: wedelolactone (30 µM), MitoTEMPOL (20 µM), Indo: indomethacin (100 µM), and PD98059 (20 µM). Data show the results of four independent experiments for each media condition, where each condition was tested in technical quadruplicate wells in each experiment.

As a first step in assessing signaling pathways associated with the POLG-variant mitogenic effect, we transfected A7r5 cells with the initial series of variant plasmids (not the P2AT2A versions) in GM or DM for 6 h and allowed cells to grow for 3 days after transfection (Figure 8A). The cell density was assessed by threshold-based nuclear counting. Drugs tested included the IKKα/ß inhibitor wedelolactone (30 µM); the mitochondria-targeted, superoxide scavenger MitoTEMPOL (20 µM); the cyclooxygenase inhibitor indomethacin (100 µM); and the ERK/MEK inhibitor PD98059 (20 µM). We normalized the cell density to the number of cells in the control-treated (no drug), WT POLG-transfected wells on each plate. In a two-factor ANOVA assessing plasmid and drug treatment as factors affecting the cell density in A7r5 grown in DM, plasmid (*p* < 0.0001) and drug treatment (*p* < 0.0001) and their interaction (plasmid x drug *p* = 0.023) were significant sources of variance. In cells grown in GM, factors such as plasmid (*p* < 0.0001) and drug (*p* < 0.012) were significant, but their interaction (*p* = 0.71) was not. In *post hoc* tests, Y955C (DM: *p* = 0.006, GM: *p* < 0.0001) and R964C (DM: *p* < 0.001, GM: *p* < 0.0001) increased the cell density (average across drug treatments). In DM conditions averaged across plasmids, all drug treatments reduced the cell density (p: < 0.0001–0.006), but in GM conditions, only PD98059 (*p* = 0.0017) reduced growth (again including all plasmid conditions). More importantly, we were interested in whether the mitogenic effects of the POLG variants were suppressed by these inhibitors. As the cell density of Y955C- and R964C-transfected wells was generally not significantly different, we compared the WT cell density to the combined measures of cell density in Y955C and R964C plasmids, which would have a greater statistical power to detect a difference and thus reduce false conclusions that there is a lack of difference (i.e., lower type II error). In normal growth media (GM) and 1% serum media (DM) with reduced growth factors, we could not detect a mitogenic effect of Y955C/R964C in cells treated with wedelolactone or MitoTEMPOL, although the lack of difference was marginal for wedelolactone in GM (Figure 8B). In cells exposed to indomethacin and PD98059, the mitogenic effect of the Y955C and R964C variants persisted, suggesting that inflammatory signaling involving cyclooxygenase and classic growth-factor-dependent activation of ERK/MEK signaling are unlikely to be driving the mitogenic response mediated by overexpression of LOF POLG variants.

## 4 Discussion

### 4.1 Main conclusions

Mitochondrial dysfunction (acquired and inherited) is known to promote cardiovascular disease, but the extent to which genetic variants in the POLG gene might contribute to vascular dysfunction is not well understood. Over 300 POLG variants are linked to a variety of multi-system neurological and muscular diseases and disorders (Chan and Copeland, 2009; Young and Copeland, 2016; Rahman and Copeland, 2019). Typically, when variants are defined as pathogenic, VUS, or non-pathogenic, this is within the context of those diseases, among which hypertension is not considered. This has two important consequences: (1) the coding of variants for pathogenicity may not predict their relationship to hypertension and (2) hypertension may be overlooked as a condition contributing to POLG-associated cardiac and neurovascular disease. It was the unusually high incidence of hypertension in a cohort of patients with CPEO (chronic progressive external opthalmoplegia) and POLG variants that motivated this study (Hannah-Shmouni et al., 2014; Pauls et al., 2020). Here, we have demonstrated that both pathogenic POLG variants and POLG VUS, whose function we assume mimics or exaggerates a homozygous genotype, promote a mitogenic effect on both actively dividing and quiescent cultured vascular smooth muscle cells and HeLa cells. In the vasculature, mitogenic stimulation of the smooth muscle could contribute to smooth muscle hypertrophy or intimal thickening. In the context of HeLa cells, a link between loss of mitochondrial DNA copy number and hyperplasia may shed light on the link between reduced mCN and cancer (reviewed in Li et al., 2022).

Our early understanding of the impacts of reduced mCN come from studies of mtDNA-depleted (rho 0) cells, which typically demonstrate growth deficit due to metabolic deficiencies when mtDNA falls below ∼25% of normal levels (Bodnar et al., 1995; Jazayeri et al., 2003). Subsequent studies of proof-reading deficient POLGs and induced mtDNA damage painted a more complex picture of the relationship between mtDNA maintenance and cell physiology. For example, the mitochondrial mutator mice, with their proof-reading-impaired POLG, exhibit multiple aspects of progeria and are prone to hypertension. Initial studies suggested that accelerated aging occurred in the absence of extensive ROS production, which is in contrast to the notion that mtDNA damage leads to a pernicious cycle of ROS-induced mtDNA damage (Trifunovic et al., 2005; Golob et al., 2015). However, a further study suggests that the mutator mouse does exhibit elevated levels of DNA damage and elevated 8-oxoguanine in urine, which has been associated with elevated ROS production (Yu et al., 2022). Nonetheless, some of the consequences of mitochondrial dysfunction beyond ROS generation include triggering the innate immune response (West, 2017), induction of pseudo-senescence-associated secretory phenotype (Wiley et al., 2016), and transitioning quiescent cells into a G-alert state (Baker et al., 2022). In the mutator mouse, the lack of POLG proofreading induces sex-dependent changes in cardiac morphology, where aged males show left ventricular thinning *versus* aged females exhibiting ventricular hypertrophy (Gorr et al., 2022). Notably, recent studies on POLG function have largely focused on the D275A proofreading deficient variant found in the mutator mouse. In contrast, few studies have examined the effects of variants in the polymerase domain, such as those that we have studied. We observed that exogenously expressing loss-of-function POLG variants in a subset of the cell population unexpectedly reduced mCN throughout the cell population and caused the release of a diffusible mitogenic signal, which suggests that mitochondrial dysfunction in a subset of cells may impact neighboring cells.

Several lines of evidence support the conclusion that cells neighboring transfected cells experienced a decrease in mCN. Indirect immunolabeling of the exogenous POLG with both Myc and FLAG-tagged epitopes confirmed that transfection efficiency of A7r5 cells was ∼10–30% of cells, and POLG::FLAG labeling agreed well with the presence of the NLS-EGFP when we transfected cells with P2AT2A constructs. This indicated that mitochondrial transfer did not lead to extensive distribution of faulty POLG. The estimation of mtDNA content in single cells by anti-dsDNA-staining did not show differences between POLG+ and POLG-cells, and these estimates of mCN were in good agreement with the absolute quantification of ddPCR. Furthermore, the rank order of mCN loss (Y955C > R964C > N1098I > R1138C) essentially agreed with the predicted pathogenicity. If, in contrast, the reduced mCN were primarily due to extensive loss of mtDNA from only the transfected cells, then the ∼40% decrease in mCN in the Y955C-transfected cultures would have required the transfected cells to be almost devoid of mtDNA, which is contradictory to our observations. Furthermore, the general agreement between ddPCR and image-based mCN assessment (see Li et al., 2018) provides reassurance that the loss of mtDNA, measured by imaging, reflects a true loss of mtDNA rather than oxidative mtDNA modification or super-packing, leading to reduced antibody binding (He et al., 2007; Di Re et al., 2009; Li et al., 2022). Notably, the N1098I and R1138C variants mediated reductions in mCN, which in some conditions were not statistically significant, but nonetheless, they mediated mitogenic effects in both A7r5 and HeLa cultures. Thus, the N1098I and R1138C variants should be further investigated to clarify pathogenic roles not only in neuromuscular disease but also in hypertension and cancer.

The recent advent of open-access machine learning and artificial intelligence-based protein structure predictors like AlphaFold presents an additional potential tool in predicting the pathogenicity of genetic variants. Our initial evaluation of AlphaFold’s ability to predict deviations of the typical orientation of POLG and DNA at the catalytic site suggested that Y955C and N1098I would cause overt structural changes. Future analyses with novel tools such as RoseTTAFoldNA and DeepPBS might provide further structural insights to understand and predict the pathogenicity of diverse POLG and POLG2 variants (Baek et al., 2024; Mitra et al., 2024).

The linear relationship between reduced culture-wide mCN and cell proliferation to the best of our knowledge is a novel observation, which warrants consideration. Specifically, we were initially concerned that this relationship simply reflected smaller cells in more dense cultures having lower mCN as mCN is reported to scale with cell size (volume) in yeast and mammalian cells (Seel et al., 2023). However, when we plotted the absolute cell density as a function of estimated mCN, the linear relationship did not persist. Furthermore, since cells were plated at different starting densities in some experiments, we were able to assess that mCN was not lower in more densely plated cultures. In contrast, we did observe an association between mCN and mitochondrial mass, as assessed by MitoTracker staining, suggesting that reduced mCN may have been related to a parallel change in mitochondrial content (e.g., reduced mitochondrial biogenesis or increased mitophagy). This was consistent with reports that increases in mtDNA copy number during cell-cycle progression require mitochondrial replication (Sasaki et al., 2017), and, by corollary, reduced mCN would coincide with mitochondrial depletion or limit mitochondrial biogenesis.

To the extent that we could distinguish portions of the cell cycle, primarily G0/G1 vs. S-phase, we could not observe any change in the cell-cycle distribution associated with the mitogenic effects of the POLG variants. In HeLa cells, the mtDNA nucleoid number is reported to double, largely during S-phase and G2/M (Sasaki et al., 2017). In A7r5, we similarly saw mCN, primarily increasing during the S-phase, but it was only by ∼20% relative to the mean of the G0/G1. If A7r5 cells do, indeed, double their mCN during cell division, this suggests that mean mCN in the G0/G1 population incorporates cells that have recently divided and those that have significantly increased mCN during G1. GM-treated A7r5 cells in the “4N” population include both diploid cells in G2/M phases and *bona fide* tetraploid cells in G0/G1, which we cannot readily distinguish when cells are freely progressing through he cell cycle. However, in DM conditions, A7r5 cells stall in the S-phase, such that most 4N cells are legitimate tetraploid cells, the same being true for a smaller population of octoploid cells. Notably, in DM conditions, 4N (tetraploid G0/G1) cells have a mean mCN that was not different from that of 2N cells, and 8N (octoploid G0/G1) cells had a mCN that was roughly two-fold rather than the four-fold greater than the diploid level. This suggested that polyploid A7r5 cells, in this study at least, were metabolically distinct or dysfunctional relative to their diploid counterparts.

Overall, the metabolic state of the A7r5 cells was minimally impacted by over-expression of the POLG variants. The observation that mitochondrial membrane potential and intracellular ROS measurements did not differ between transfected cells and untransfected neighbors was largely consistent with a parallel lack of difference in mCN. Rather, mitochondrial membrane potential was reduced in the 8N population, and ROS production was elevated in the 4N and 8N populations, suggesting that the polyploid cells were metabolically unhealthy, possibly due to a lack of mtDNA. However, since the fraction of 4N and 8N cells did not change with over-expression of POLG loss-of-function variants, this was unlikely to account for the mitogenic effects of the POLG variants. The Seahorse assay confirmed that no change in oxidative metabolism was detectable in DM-treated conditions, whereas in GM-treated cells, a very modest decrease in basal respiration, estimated ATP production, proton-leak, maximal respiration, and non-mitochondrial respiration was observed and consistent with greater loss of mtDNA in the actively dividing cells. *A priori*, one might expect that reduced respiration would suggest that cultures would exhibit reduced growth, similar to the phenotype of mtDNA-depleted cells (Bodnar et al., 1995; Jazayeri et al., 2003). However, the modest metabolic effect that we observed appears to be outweighed by a parallel mitogenic stimulation.

We turned to live-cell imaging with the goals of assessing growth rates and to assess how transfected cells behaved relative to untransfected neighbors. SiR-DNA is a far-red nuclear stain that is compatible with live-cell imaging with minimal toxicity (Lukinavičius et al., 2015), and Spy650-DNA is its successor with enhanced cell retention. We titrated dye concentrations to use the lowest concentration that permitted clear nuclear labeling to minimize the toxicity. Our custom-written Fiji macros enabled efficient, largely hands off, StarDist segmentation of nuclei in 210-frame movies from 80 wells per 96-well plate. In untransfected A7r5 cells, this approach produced growth rates that were highly consistent over a range of cell plating densities (Supplementary Figure 5), which would provide a more robust metric of growth compared to endpoint growth analyses. However, A7r5 cells did not tolerate the combined stress of transfection and prolonged imaging. We used HeLa cells stably expressing H2B-RFP in pilot studies of cell segmentation, and these cells both tolerated post-transfection and multi-day imaging and revealed that the mitogenic effect of the POLG variants was not specific to smooth muscle cells. Thus, we confirmed that conditioned media from transfected HeLa cells contained a mitogenic signal that stimulated growth in naïve cultures.

Identification of the specific mitogenic factor and mechanisms inducing reduced mCN was beyond the scope of the current study but will be further assessed in future studies. Our preliminary pharmacological analysis suggests that the downstream signaling pathways likely involve pathways associated with nuclear factor kappa-light-chain-enhancer of activated B cells (NF-kB) and possible mitochondrial ROS generation, based on wedelolactone and MitoTEMPOL suppressing the mitogenic effects of the Y955C and R964C variants. If the POLG variants result in secretion of incompletely replicated or faulty mtDNA from cells, it is possible that toll-like receptors recognizing the cell-free mtDNA as bacterial DNA could initiate an inflammatory mitogenic response. Activation of vascular TLR9 receptors is reported to mediate hypertensive effects in response to circulating mtDNA in rats (McCarthy et al., 2015; 2018) and inflammatory pulmonary endothelial damage in bacteria-induced inflammation (Simmons et al., 2013; Kuck et al., 2015). Further mechanistic clues might come from *Polg* ^D257A^ mice, whose impaired POLG exonuclease function increases innate immune responses by increasing the macrophage expression of inflammatory cytokines and interferon-1 (see pre-print of VanPortfliet et al., 2024). By corollary, fibroblasts generated from patients with a compound heterozygous p.Trp748Ser and p.Arg709* or p.Thr251Ile, p.Pro587Leu, and p.Glu1136Lys POLG variants exhibited reduced cell growth when mCN was rescued by nucleoside supplementation (Dombi 2024 Front Cell Dev Biol). Furthermore, given that specific POLG variants are linked to diverse diseases, one might assume that the cellular phenotype associated with diverse variants will differ. To this end, cortical organoids derived from induced pluripotent stem cells with compound p.Ala467Thr and p.Pro589Leu variants from patients with Alpers’ disease exhibit enhanced ROS and complex I function, which is in contrast to the impacts of the variants characterized (Hong and Liang 2024 Int J Biol Sci). As with other models involving variants being present throughout the cell population, the involvement of diffusible factors mediating cellular phenotypes may not be immediately obvious, but it is promising that in this later example, mitochondrial dysfunction was rescued by NAD^+^ supplementation.

### 4.2 Limitations

Several limitations should be considered when interpreting our results. AlphaFold Server is currently one of the best protein structure prediction tools, but it is, in the end, a prediction, and the reported POLG/DNA interactions were based on overt structure changes. Future analyses should include additional measures of the POLG/DNA interaction surface and detailed analyses of interactions strengths. These *in silico* analyses will ideally be paired with future *in vitro* analyses of the effects of the novel variants on polymerase catalytic activity and accuracy. Our measures of mitochondrial membrane potential were population-based, and analyses of cells sequentially treated with oligomycin and FCCP might provide a more sensitive measure. While the concentration of TMRM used was well below the concentrations at which the dequenching mode occurs ( > 300 nM in preliminary experiments), we did use MitoSOX at concentrations (5 µM) that lead to notable nuclear localization (Roelofs et al., 2015). Notably, this concentration of MitoSOX is often used in analyses of ROS measures by FACS or FLOW cytometry, in which dye localization is not observed, but pharmacological and molecular interventions are consistent with a mitochondrial source of ROS (Gerner et al., 2019; Joe et al., 2019; Little et al., 2020; Prasad et al., 2020; Yang et al., 2021). Given the ability of MitoTEMPOL to suppress the mitogenic effects of the Y955C and R964C variants, it is possible that we were unable to detect modest ROS elevations. Furthermore, in Seahorse experiments, the responses measured upon FCCP addition were below basal values, limiting the accuracy of the uncoupled respiration values. This may be characteristic of A7r5 cells, as others have reported similar observations in A7r5 cells using 1 µM FCCP (Pace et al., 2016). However, this is unlikely to have affected the over-arching conclusions that any changes in mitochondrial metabolism were modest. The persistent expression of the native POLG in the cells could be expected to maintain some level of accurate mtDNA, thereby underestimating the impact of the POLG variants studied. To this end, astrocytes generated from human induced pluripotent stem cells harboring a homozygous p.W748S or compound p.A467T/W748S variants exhibited ∼40% reductions in mCN and reduced mitochondrial membrane potential, ATP production, and respiratory chain activity (Liang et al., 2024). Interestingly, despite reduced mitochondrial metabolism, these astrocytes showed increased viability and proliferation akin to the phenotype reported herein, consistent with our conclusion that the mitogenic effect of POLG variants may not be dependent on impaired mitochondrial metabolism. For experiments involving drug treatments, drugs were typically added as 1000x dilutions of stocks made in water, DMSO, or ethanol. Vehicle controls were not included, as we have typically found the A7r5 cells to be unaffected by 0.1% DMSO or 0.1% ethanol. Finally, based on our current data, it is not clear whether parallel changes in mitochondrial biogenesis and mitophagy could have limited our ability to detect the effects of the variants studied or if the effects were observed with the result of retrograde nuclear signaling or altered expression of mitochondrial signaling peptides such as humanin or MOTS-C.

### 4.3 Impact on the field

The key novel finding of our study is that a range of POLG variants that include known pathological variants and those predicated to be non-pathogenic, when present as mono-alleles, all exhibited a mitogenic effect that was consistent with predicted rank-order loss-of-function. This mitogenic effect was observed even when changes in mitochondrial DNA copy number were below the limit of detection (∼10% reduction), for example, in the case of the N1098I variant. Our current evidence indicates that the mitogenic signal was not cell-autonomous but involved a secretory factor, the identification of which could provide a therapeutic avenue for future assessment in the context of both hypertension and predisposition to cancer. Further studies will be required to determine if the novel variants studied here also impacted the mutation rate or levels of mtDNA linear fragments in the cells and any downstream impacts thereof. Moreover, it is important to note that mitogenic signaling in the context of the intact vascular media might result in polyploidization rather than proliferation.

## Data Availability

The raw data supporting the conclusions of this article will be made available by the authors, without undue reservation.
