# Supplementary material for: Loss-of-function mitochondrial DNA polymerase gamma variants cause vascular smooth muscle cells to secrete a diffusible mitogenic factor
Source: Front Physiol. 2025 Feb 17;15:1488248. doi: 10.3389/fphys.2024.1488248 (PMC11873068; doi:10.3389/fphys.2024.1488248)
Supplement: Supplementary file 1 [file Presentation1.pdf]

Supplemental Figure 1

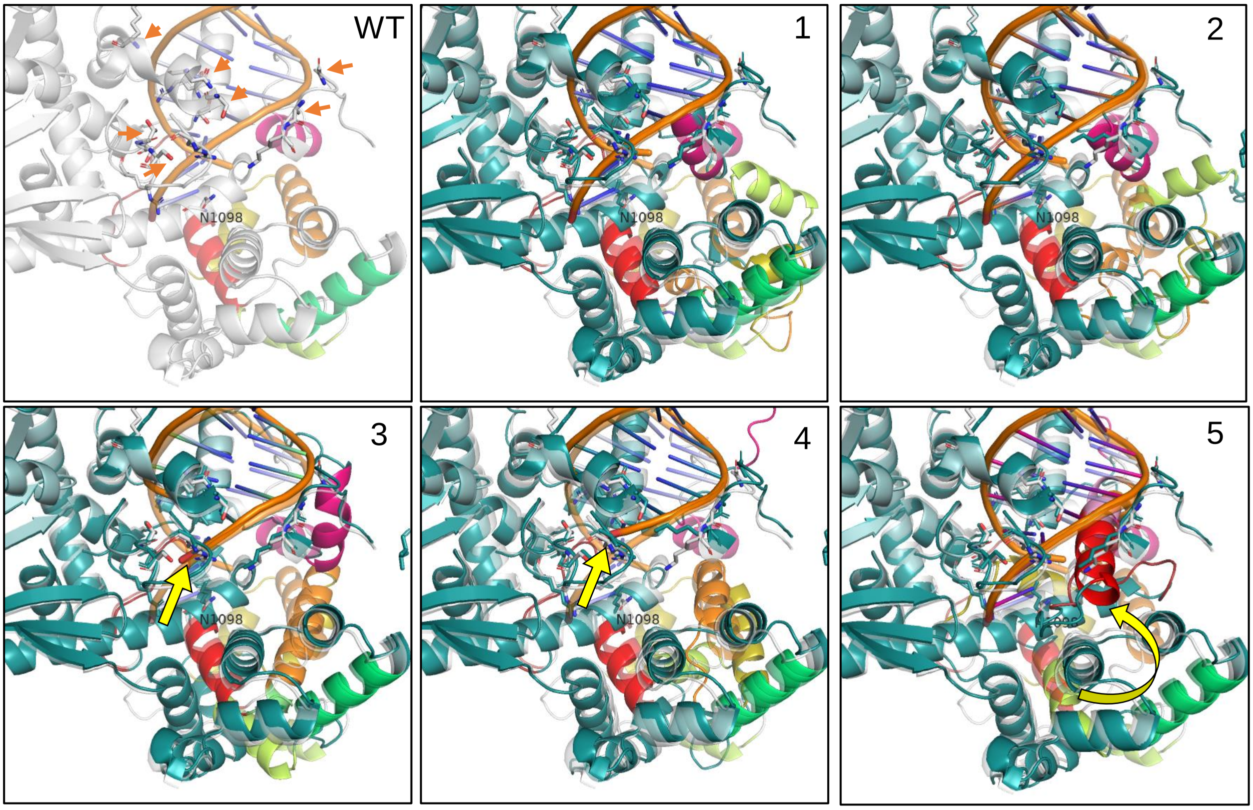

AlphaFold modelling of POLG N1098K.

"WT" panel shows AlphaFold model of POLG and DNA highlighting location of N1098 and residues (orange arrows) within 4 of the DNA surface (306, 309, 763, 767, 768, 799, 803, 806, 849, 853, 861, 863, 869) shown with residues as sticks. Panels 1-5 show modelled orientation of POLG catalytic site and DNA for the N1098K variant. Models 3 and 4 show DNA not seating as deep in the catalytic site, as shown with yellow arrows. Model 5 shows the 1056-1066 helix swinging (yellow arrow) into the active site as models predicted for Y955C and N1098I variant.

Supplemental Figure 2

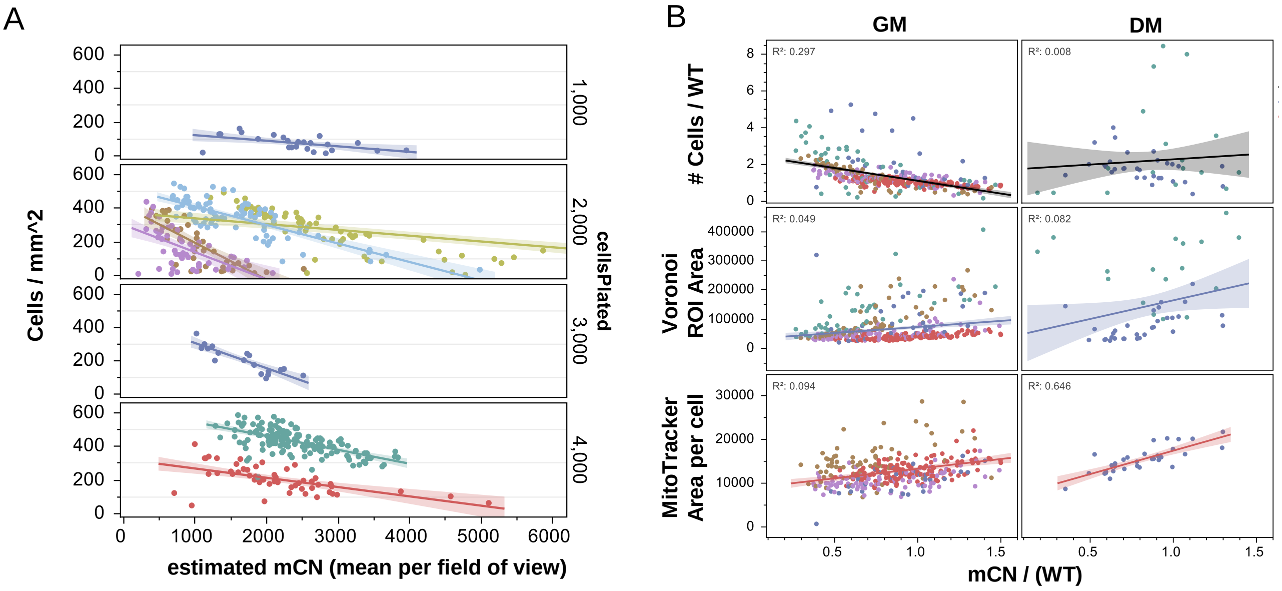

Correlates of imaged mitochondrial DNA copy number.

(A) Estimated mitochondrial DNA copy number regression with absolute cell density by 96-well plate shows that cells plated at higher density do not on average have lower mCN. Colors indicate independent platings. (B) Comparison of correlation of normalized mCN with normalized cell density, size of the Voronoi defined cellular regions of interest, and mean area (# of pixels) of MitoTracker Orange above threshold as a measure of mitochondrial mass.

Supplemental Figure 3

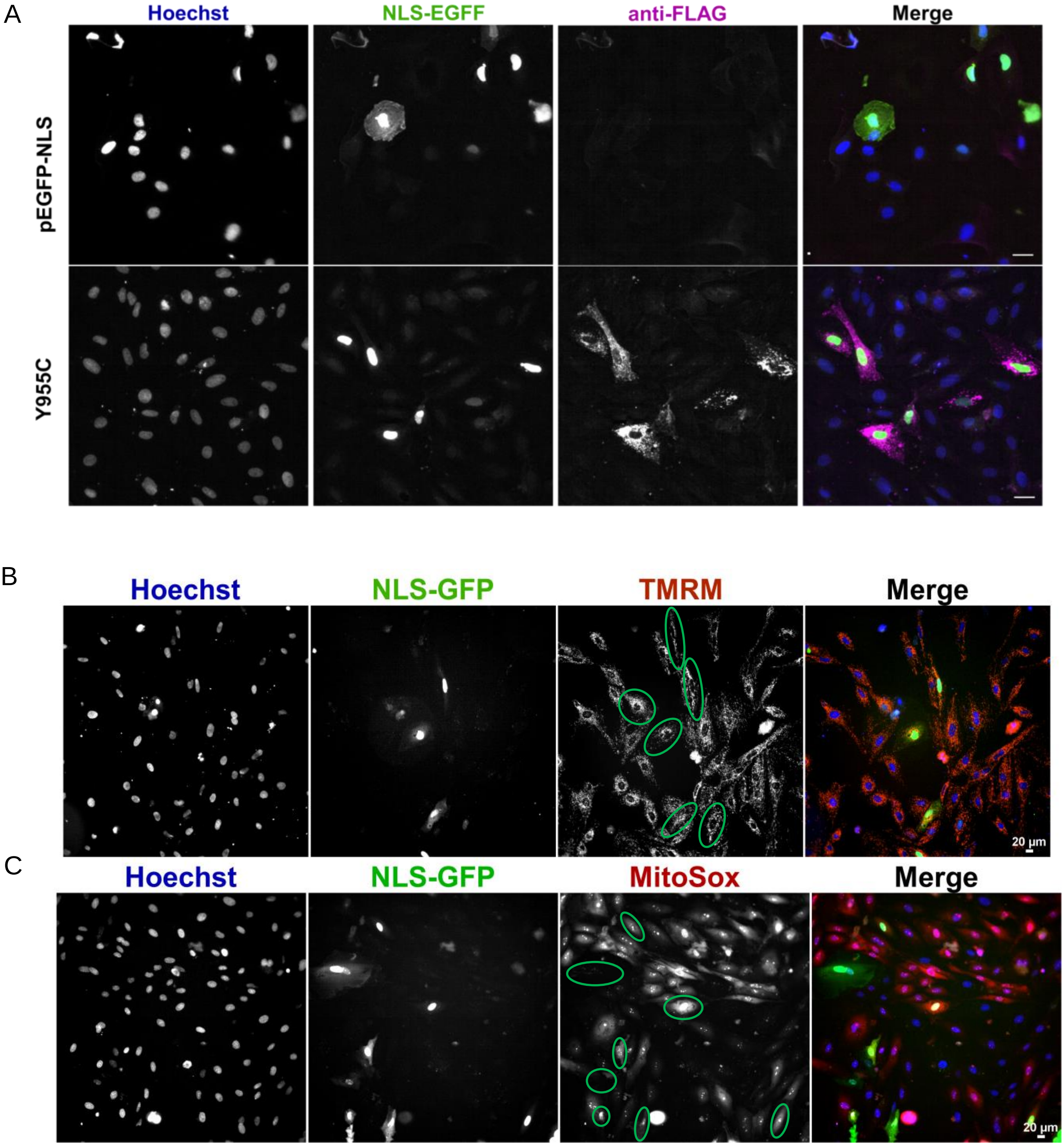

Co-detection of FLAG-tagged POLG and NLS-EGFP and Mitochondrial membrane potential and ROS in Y955C-transfected cells versus there neighbours.

(A) Exemplar imaged of A7r5 cells were transfected with pEGFP3xNLS (upper) or pPOLG::P2AT2A::EGFP3xNLS (lower: Y955C variant shown) and stained with anti-FLAG antibody (1:400) and AlexaFluor® 647 Goat anti-mouse IgG_1 (1:500) and counter-stained with Hoechst 33342. Grey scale images are evenly scaled for upper and lower panels. No FLAG labelling was evident in the absence of the POLG vector. Scale bar is 50 µm. (B&C). These images complement Figure 6A and 6F, showing (B) TMRM and (C) MitoSox staining in A7r5 transfected with pPOLG(Y955C):P2AT2A:EGFP3xNLS

Supplemental Figure 4

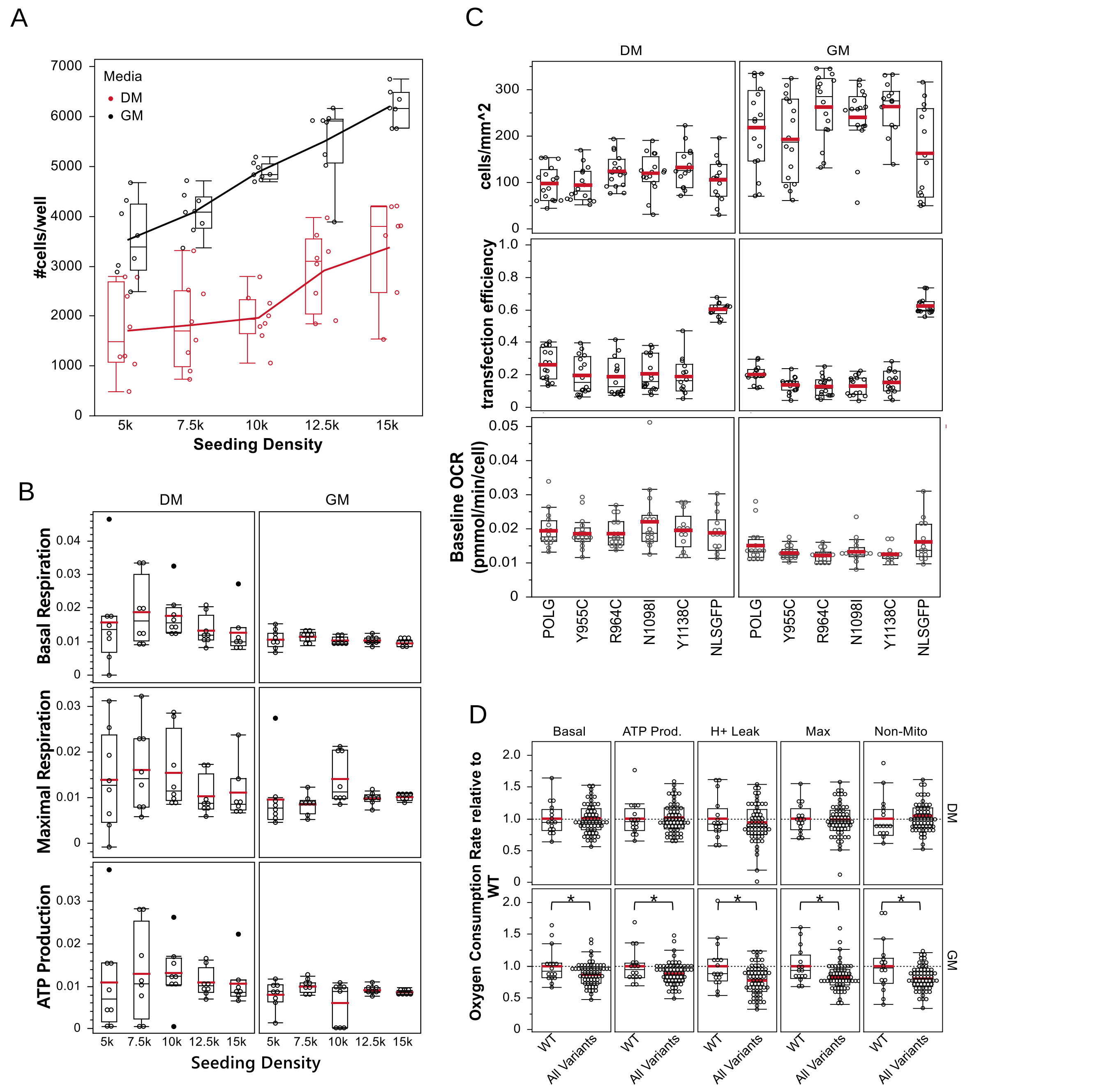

Seahorse optimization, and additional quantitative analyses

(A) A7r5 cells were plated at 5000-15000 cells per well in Seahorse plates. After 24 hours, media was changed to GM or DM and cells grew for 48 h before being fixed, Hoescsht-stained and imaged on the Nikon TiE and counted using StarDist. (B) OCR (pmol/min/cell) was similar over the range of cell plating densities in DM and GM, with reduced variance at higher plating density. (C) (Upper) Example of cell density assessment after Seahorse measurements in plates transfected with pPOLG:P2AT2A:EGFP3xNLS and variants. Data points are fields of view, with means trending higher in cultures transfected with variants. (Middle) Transfection efficiency determined from NLS-GFP. (Lower) Baseline OCR/cell in GM and DM conditions. (D) OCR normalized per cell and to WT plate-wise mean for each media condition where POLG variants are combined (Y955C, R964C, N1098I, and R1138C). Asterisks represent significance ($p<0.05$).

Supplemental Figure 5

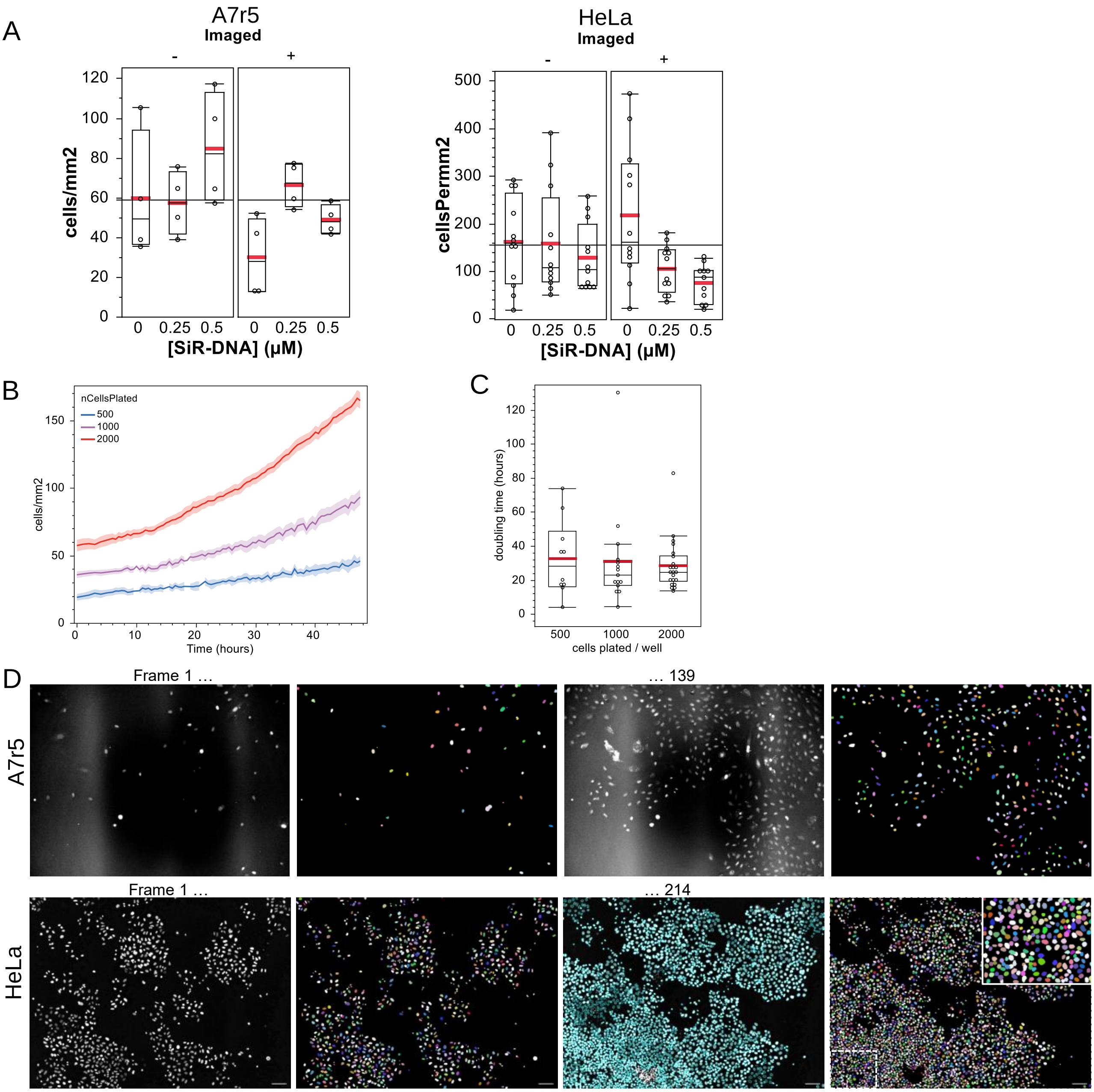

Cell Growth during IncuCyte Imaging and Impact of Far-Red Dyes on Growth.

(A) A7r5 and HeLa cells were plated at 29 cells/mm^2 (1000 cells/well) on 96-well plates and 48h later were loaded with 0, 0.25 or 0.5 µM SiR-DNA. Parallel wells were imaged or not imaged for 48 hrs. Wells were imaged with filter sets for orange to image the H2B-RFP in HeLa cells (A7r5 also imaged for control) and near infrared to image SiR-DNA. At the end of 48 hours, media was replaced in all wells with media containing 0.5 µM SiR-DNA and cells were imaged for an additional 5-24 hours. Summary data is shown for cell density measured at the second 30-minute time point. A7r5 cells showed little sensitivity to SiR DNA at 0.25 – 0.5 µM. Imaging HeLa with 0.25 and more so 0.5 µM SiR DNA showed moderate growth inhibition. (B) Example growth curves of cells labelled with 0.25 µM SiR-DNA when plated at varying densities. (C) Growth curve analysis indicated that population doubling times were consistent over a wide range of plated cell density. (D) StarDist Versatile Fluorescent Nuclei ROI generation overlay for A7r5 and HeLa cells labelled with 0.5 µM SiR-DNA during long-term live cell imaging in the IncuCyte.
